# Supplementary material for: Dielectrophoretic bead-droplet reactor for solid-phase synthesis
Source: Nat Commun. 2024 Jul 22;15:6159. doi: 10.1038/s41467-024-49284-z (PMC11263596; doi:10.1038/s41467-024-49284-z)
Supplement: Supplementary file 1 — Supplementary Information [file 41467_2024_49284_MOESM1_ESM.pdf]

Supplementary Information: Dielectrophoretic Bead-Droplet Reactor for Solid-Phase Synthesis

Punnag Padhy[1*], Mohammad Asif Zaman[1], Michael Anthony Jensen[2,3*], Yao-Te Cheng[1], Yogi Huang[4], Mo Wu[1], Ludwig Galambos[1], Ronald Wayne Davis[2,3,5] and Lambertus Hesselink[1*]

[1]Department of Electrical Engineering, Stanford University, Stanford, CA, 94305, U.S.A

[2]Stanford Genome Technology Center, Stanford University, Palo Alto, CA, 94304, U.S.A

[3]Department of Biochemistry, Stanford University, Stanford, CA, 94305, U.S.A

[4]Department of Chemical Engineering, Stanford University, Stanford, CA, 94305, U.S.A

[5]Department of Genetics, Stanford University, Stanford, CA, 94305, U.S.A

*punnag@stanford.edu, m.a.jensen@stanford.edu, hesselink@ee.stanford.edu

Contents

1. **Supplementary Note 1:** Electrohydrodynamic simulation of the encapsulation and ejection of the bead by the droplet.
2. **Supplementary Note 2:** Analytical description of the dielectrophoretic force and the emergence of Clausius-Mossotti factor.
3. **Supplementary Note 3:** Potential energy-based description of the encapsulation and ejection process.
4. **Supplementary Note 4:** Role of geometrical and fluidic properties on the encapsulation and ejection process.
5. **Supplementary Note 5:** Particle tracking simulation of bead motion in synthesis columns on introduction of reagents.
6. **Supplementary Note 6:** Electric field driven enhancement of reagent concentration within droplet.
7. **Supplementary Figure 1:** Device Fabrication.
8. **Supplementary Figure 2:** Experimental Setup.
9. **Supplementary Figure 3:** Sample Mounting.
10. **Supplementary Figure 4:** Electrohydrodynamic simulation setup.
11. **Supplementary Table 1**: Table of all the geometric and material parameters used in electrohydrodynamic simulations and energy calculations.
12. **Supplementary Figure 5:** Electric field response of beads and droplet.
13. **Supplementary Figure 6:** Change in the droplet dimension with the encapsulation of the bead into the droplet.
14. **Supplementary Figure 7:** Fluorescence images and intensity data for enzymatic coupling and control experiment.
15. **Supplementary Figure 8:** Benchtop synthesis reaction.
16. **Supplementary Figure 9:** Fluorescence data for coupling and control reactions implemented in synthesis columns (benchtop reactions).

17. **Supplementary Figure 10:** Image processing of benchtop reaction snapshots to extract fluorescence data from beads.
18. **Supplementary Table 2:** Statistical significance testing for reaction fidelity enhancement using Welch t-test on fluorescence intensity data.
19. **Supplementary Table 3:** Statistical significance testing for reaction fidelity enhancement using Welch t-test on ranks of fluorescence intensity data.
20. **Supplementary Figure 11:** Reagent access to bead surfaces in various packing configurations and in DBDR.
21. **Supplementary Figure 12:** Turbulent flow driven particle tracking simulation setup.
22. **Supplementary Figure 13:** Role of diffusion in reagent access to bead surfaces in stacked bead configurations and in DBDR.
23. **Supplementary Figure 14:** Simulation for concentration enhancement of ionic species on the bead surface.
24. **Supplementary Table 4:** Summary of parameters used in electric field driven ion transport modelling.
25. **Supplementary Figure 15:** Concentration enhancement of ionic reacting species during the A.C. supply cycle.
26. **Supplementary Figure 16:** Concentration enhancement of ionic reacting species during the A.C. supply cycle with varying supply frequency.
27. **Supplementary Figure 17:** Concentration enhancement of ionic reacting species during the A.C. supply cycle with varying supply voltage.

Supplementary Note 1: Electrohydrodynamic simulation of the encapsulation and ejection of the bead by the droplet

We modeled the electric field-driven engulfing of the bead by the droplet and its subsequent ejection by coupling the Navier Stokes equation for incompressible fluids[S1] (eq. S1) with the charge continuity equation of electrodynamics[S1-S3] (eq. S2). The spatially varying electric field due to suitably defined electrodes exerts non-uniform pressure on the fluidic interface resulting in a net force on the aqueous reagent droplet suspended in a medium of silicone oil[S5-S7] with a kinematic viscosity of 1cSt. The electric force exerted on the fluidic interface is given by the Maxwell Stress Tensor[S2-S7] (eq. S3). This electric force drives fluid flow as per the Navier Stokes equation[48] (eq. S1). As a result, the fluidic interface shifts with time. The shift in fluidic interface with time is tracked by the phase-field method[S8] (eq. S4). This shifting fluidic interface is fed back into the electrical charge continuity equation (eq. S2) as a change in the material boundary and hence a change in the electric boundary condition. Mimicking experimental observations (Fig. 2(a) in the main text) the bead was kept stationary in the simulations. This also reduces the number of moving components and greatly simplifies the simulation while keeping the essential physics underlying the process largely intact. Furthermore, we employ an axis-symmetric model for our simulation to reduce computational resource requirements (Supplementary Figure 4) while focusing on the essential physical principles underlying the process.

The Navier Stokes equations are given by[S1]:

$$\rho \frac{\partial \vec{u}}{\partial \mathrm{t}} + \rho(\vec{u} \cdot \vec{\nabla})\vec{u} = \vec{\nabla} \cdot \left[-p\overleftrightarrow{I} + \overleftrightarrow{K}\right] + \vec{f}_{electric} \qquad \text{(eq. S1a)}$$

$$\rho\vec{\nabla} \cdot \vec{u} = 0 \qquad \text{(eq. S1b)}$$

$$\overleftrightarrow{K} = \mu\left\{\left(\vec{\nabla}\vec{u}\right) + \left(\vec{\nabla}\vec{u}\right)^T\right\} \qquad \text{(eq. S1c)}$$

$$\vec{f}_{electric} = \vec{\nabla} \cdot \overleftrightarrow{T}_{electric} \qquad \text{(eq. S1d)}$$

Here ρ is density of the fluid, $\vec{u}$ is velocity of fluid flow, p is pressure, $\overleftrightarrow{I}$ is the identity tensor, $\overleftrightarrow{K}$ is the viscous stress tensor, $\vec{f}_{electric}$ is the electric force density, μ is the dynamic viscosity of fluid and $\overleftrightarrow{T}_{electric}$ is the electric component of Maxwell Stress Tensor (MST). The electric charge continuity equations can be written as[S2,S3]:

$$\vec{\nabla} \cdot \vec{J} = -\frac{\partial \rho_q}{\partial t} \qquad \text{(eq. S2a)}$$

$$\overleftrightarrow{J} = \sigma\vec{E} \qquad \text{(eq. S2b)}$$

$$\vec{E} = -\vec{\nabla}V \qquad \text{(eq. S2c)}$$

$$\vec{D} = \varepsilon_0 \varepsilon \vec{E} \quad \text{(eq. S2d)}$$

$$\sigma = Vf_r\sigma_r + Vf_o\sigma_o \text{ and } \varepsilon = Vf_r\varepsilon_r + Vf_o\varepsilon_o \quad \text{(eq. S2e)}$$

Here $\vec{J}$ is the current density, ρ_q is the charge density, σ is the electrical conductivity, $\vec{E}$ is the electric field, $\vec{D}$ is the electric displacement, ε_0 is the permittivity of free space, ε is the relative permittivity, V is the electric potential, $Vf_{r/0}$ represents the volume fraction of the reagent droplet/oil medium, $\sigma_{r/o}$ and $\varepsilon_{r/o}$ represent the electrical conductivity and the electrical permittivity of the reagent droplet/oil medium respectively. The Maxwell Stress Tensor can be written as[S2-S7]:

$$\overleftrightarrow{T} = \vec{E} \otimes \vec{D} - \frac{1}{2}\left(\vec{E} \cdot \vec{D}\right) \quad \text{(eq. S3)}$$

Here $\otimes$ represents outer product of two vectors. The phase field equations for two phase flow can be written as[S8]:

$$\frac{\partial \phi}{\partial \mathrm{t}} + \vec{u} \cdot \vec{\nabla}\phi = \vec{\nabla} \cdot \frac{3\chi\epsilon_{pf}}{2\sqrt{2}}\vec{\nabla}\psi \quad \text{(eq. S4a)}$$

$$\psi = -\vec{\nabla} \cdot \epsilon_{pf}^2 \vec{\nabla}\phi + (\phi^2 - 1) \quad \text{(eq. S4b)}$$

$$\vec{f}_{st} = \frac{3\gamma}{2\sqrt{2}\epsilon_{pf}}\psi\vec{\nabla}\phi \quad \text{(eq. S4c)}$$

$$Vf_o = \frac{1-\phi}{2} \text{ and } Vf_r = \frac{1+\phi}{2} \quad \text{(eq. S4d)}$$

Here ϕ represents the phase field variable which is -1 in the suspension medium and 1 in the reagent droplet and transitions from -1 to 1 at the droplet-medium interface. The phase field method models the interface as a transition region of non-zero thickness over which the two fluids mix with varying volume fractions Vf_r and Vf_o. ϵ_{pf} is the interfacial thickness parameter which determines the stiffness of the transition from the reagent phase ($\phi = 1$) to the oil phase ($\phi = -1$). It should be small enough to maintain the sharpness of the interface and capture the physics accurately but large enough to prevent the finite mesh size from causing numerical instabilities. χ is called the mobility tuning parameter. It should be large enough to accurately track the shift in the interface position with fluid flow while still being small enough to give a sharp enough of an interface. γ is the surface tension coefficient.

As the droplet moves under the influence of the applied electric stress (eq. S1d and eq. S3) as per the Navier Stokes equation (eq. S1a-c) the spatial position of the droplet-medium interface evolves. The resultant evolution of the fluidic interface was tracked using the phase field method (eq. S4). The changing boundary was reflected as a change in the spatial profile of the electrical conductivity and relative permittivity (eq. S2d) which in turn modified the solution of the charge

continuity equation (eq. S2a). This again results in a change in the force acting on the droplet (eq. S1d and eq. S3). This system of coupled equations was solved using COMSOL Multiphysics which is a commercially available finite element method-based simulation package. The user defined material properties used in the simulation are summarized in Supplementary Table 1.

Supplementary Note 2: Analytical description of the dielectrophoretic force and the emergence of Clausius-Mossotti factor

A charge neutral dielectric particle gets electrically polarized under the influence of an external applied electric field[S2-S7]. If the electric field is spatially uniform, the induced opposite charges experience equal and opposite forces due to the polarizing field which results in a net zero force on the particle. However, if the electric field is spatially varying, the induced opposite charges experience unequal magnitude of forces in opposite directions[S2-S7]. This results in a net non-zero force acting on the polarized particle dielectric particle which is called the dielectrophoretic force ($\vec{F}_{DEP}$). In its most general form this force can be expressed in terms of the electric component of the Maxwell Stress Tensor as[S2-S7]:

$$\vec{F}_{DEP} = \iiint \vec{f}_{electric} dV = \iiint \vec{\nabla} \cdot \overleftrightarrow{T}_{electric} dV = \iint \overleftrightarrow{T}_{electric} \cdot \overrightarrow{dA} \qquad \text{(eq. S5)}$$

Although MST is a mathematically elegant representation of the electric stress acting on the dielectric particle in terms of field quantities which is well suited for numerical computations, the physical picture of the interaction of the polarized particle with the electric field is not obvious from this representation. A more insightful representation of $\vec{F}_{DEP}$ in terms of the interaction of the induced multipoles of the polarized particle with the corresponding terms in the multipolar expansion of the electric field is[S7]:

$$\vec{F}_{DEP} = \sum_n \frac{1}{n!} \bar{\bar{p}}_n [\cdot]^n \left(\vec{\nabla}\right)^n \vec{E} \qquad \text{(eq. S6)}$$

Here $\bar{\bar{p}}_n$ is the electric field induced dyadic tensor moment of order n. It is given by:

$$\bar{\bar{p}}_n = \frac{4\pi\varepsilon_0 \tilde{\varepsilon}_o R_p^{2n+1}}{(2n-1)!!} K_n \left(\vec{\nabla}\right)^{n-1} \vec{E} \qquad \text{(eq. S7a)}$$

$$K_n = \frac{\tilde{\varepsilon}_p - \tilde{\varepsilon}_o}{n\tilde{\varepsilon}_p + (n+1)\tilde{\varepsilon}_o} \qquad \text{(eq. S7b)}$$

$$(2n-1)!! = (2n-1) \times (2n-3) \times (2n-5) \ldots\ldots\ldots\ldots..5 \times 3 \times 1 \qquad \text{(eq. S7c)}$$

$$\tilde{\varepsilon}_{p/o} = \varepsilon_{p/o} + j\frac{\sigma_{p/o}}{\varepsilon_0 \omega_s} \qquad \text{(eq. S7d)}$$

Here $\tilde{\varepsilon}_o$ is the complex permittivity of the suspension medium which in our case is silicone oil, $\tilde{\varepsilon}_p$ is the complex permittivity of the dielectric particle which in our case can be either the bead or the droplet, R_p is the radius of the dielectric particle, and $\omega_s = 2\pi f_s$. In this manuscript the particle can either be the bead or the droplet. So, $R_p = R_{b/d}$. Here f_s is the A.C. supply frequency. For most order of magnitude estimates of the dielectrophoretic force, the dipolar ($n = 1$) component of the expansion in eq. S6 sufficient. Using eq. S7a and b in eq. S6 this dipolar estimate of the dielectrophoretic force is given by:

$$\vec{F}_{DEP} \approx \left(\vec{p} \cdot \vec{\nabla}\right)\vec{E} = 2\pi\varepsilon_0\varepsilon_o R_p^3 K_1 \vec{\nabla}\left|\vec{E}\right|^2 \qquad \text{(eq. S8)}$$

Here, $\vec{p} = \bar{\bar{p}}_1$ is the dipole moment of the particle and K_1 is the well-known Clausius-Mossotti factor. As is obvious from eq. S8,

$$\vec{F}_{DEP} \propto K_1 R_p^3 \quad \text{(eq. S9)}$$

The Clausius-Mossotti factors for the reagent droplet, water droplet and the bead suspended in the silicone oil medium are shown in Supplementary Figure 5a. Relevant material parameters are provided in Supplementary Table 1. The larger size of the droplet also contributes significantly to the much larger $\vec{F}_{DEP}$. As a result, when multiple droplets are dispensed into the reaction chamber the primary electrical response even at much smaller voltages ($\approx 20\ V$) are droplet mergers which hinders the primary process of bead encapsulation and ejection (Supplementary Figure 5b).

Supplementary Note 3: Potential energy-based evaluation of the engulfing and ejection process

The Gibbs free energy of the fluidic interfaces is given by[S1,S2,S9]:

$$U_{IT} = \gamma_{ow}A_{ow} + \gamma_{os}A_{os} + \gamma_{ws}A_{ws} \quad \text{(eq. S10)}$$

The total surface area of the bead is a constant (A_s) (Supplementary Figure 6). So,

$$A_s = A_{os} + A_{ws} \quad \text{(eq. S11)}$$

The contact angle that a reagent droplet forms on the surface of the streptavidin coated bead in a medium of silicone oil is given by[S1,S2,S9]:

$$\cos\theta = \frac{\gamma_{os} - \gamma_{ws}}{\gamma_{ow}} \quad \text{(eq. S12)}$$

Using eq. S12 and eq. S11 in eq. S10 we obtain:

$$U_{IT} = \gamma_{os}A_s + \gamma_{ow}(A_{ow} - \cos\theta\, A_{ws}) \quad \text{(eq. S13)}$$

The first part of the right side of eq. S13 is a constant. Therefore,

$$\Delta U_{IT} = \gamma_{ow}(\Delta A_{ow} - \cos\theta\, \Delta A_{ws}) \quad \text{(eq. S14)}$$

Specifically for the system under consideration with $R_b = 3\ \mu m$ (radius of bead) and $R_d = 25\ \mu m$ (radius of droplet), as the droplet completely engulfs the bead starting from separate bead and droplet (Supplementary Figure 6) we have:

$$\Delta A_{ws} = 4\pi \times 3^2 \mu m^2 \quad \text{(eq. S15)}$$

As the droplet engulfs the bead its volume remains constant and therefore its surface area with the surrounding medium increases. This can be expressed as follows (Supplementary Figure 6):

$$\frac{4}{3}\pi(R_d + \Delta R_d)^3 = \frac{4}{3}\pi R_d^3 + \frac{4}{3}\pi R_b^3 \quad \text{(eq. S16a)}$$

$$\Delta A_{ow} = 4\pi(R_d + \Delta R_d)^2 - 4\pi R_d^2 \quad \text{(eq. S16b)}$$

From eq. S16a $\Delta R_d = 0.01\mu m$. Therefore, $\Delta A_{ow} = 4\pi \times 50.0144 \times 0.0144\mu m^2$. Comparing the terms on the right side of eq. S14 we see:

$$\frac{-\cos\theta \Delta A_{ws}}{\Delta A_{ow}} = 10.82 \quad \text{(eq. S17)}$$

Therefore, we neglect ΔA_{ow} in our calculations. So, we assume:

$$\Delta U_{IT} \approx -\gamma_{ow} \cos\theta \, \Delta A_{ws} \qquad \text{(eq. S18)}$$

As the droplet moves the electrical energy of the system changes. That means the electrical energy stored in the circuit changes[S6].

$$\Delta U_E = -\frac{\Delta(QV_s)}{2} \qquad \text{(eq. S19)}$$

Here Q is the charge on the electrode and V_s is the supply voltage on it. The total change in the energy of the system as the droplet encapsulates and ejects the bead is given by:

$$\Delta U = \Delta U_{IT} + \Delta U_E \qquad \text{(eq. S20)}$$

As the droplet moves its center of mass shifts which was evaluated using the following equation for all z such that $\phi > 0$ in the simulation.

$$z_d = \frac{\sum \rho z \Delta V}{\sum \rho \Delta V} \qquad \text{(eq. S21)}$$

The center of mass of the bead is fixed (z_b) in our simulations. The distance between the center of masses is then evaluated as:

$$\Delta z_{CM} = z_d - z_b \qquad \text{(eq. S22)}$$

The change in energies is plotted against Δz_{CM} in Fig. 2c and d of the main text.

The mean capillary force acting on the bead-droplet system as the bead moves across the interface from being completely ejected just outside the droplet to completely encapsulated just inside the droplet (Supplementary Figure 6) is given by:

$$\langle |\vec{F}_{IT}| \rangle = \frac{|\Delta U_{IT}|}{2R_b} \approx \frac{\gamma_{ow} \cos\theta \Delta A_{ws}}{2R_b} = \frac{4\pi R_b^2 \gamma_{ow} \cos\theta}{2R_b} = 2\pi R_b \gamma_{ow} \cos\theta \qquad \text{(eq. S23)}$$

Supplementary Note 4: Role of geometrical and fluidic properties on the encapsulation and ejection process.

The analytical models in sections 2 and 3 give an understanding of the impact of the various geometrical and material parameters of the bead-droplet-medium system on the encapsulation and ejection process. In this section we explicitly analyze these factors by running simulations with varying parameters backed by experimental measurements of interfacial fluidic properties.

A smaller droplet experiences a smaller dielectrophoretic force (eq. S9) for the same voltage supply ($|\vec{E}|^2$ in eq. S8 is directly proportional to $|V_s|^2$). Therefore, it requires a larger supply voltage to provide large enough of a dielectrophoretic force to overcome the interfacial tension force ($\propto \gamma_{ow} R_b$) at the bead-droplet-medium interface to encapsulate the bead. As we can see in Fig. 3a of the main text, the larger droplet ($R_d = 50\ \mu m$) can encapsulate the bead at a supply voltage of $85\ V$. However, the smaller droplet ($R_d = 20\ \mu m$) cannot encapsulate the bead at the same voltage (Fig. 3b of main text). To compensate for the reduced droplet size, a much higher voltage of $135\ V$ is required to overcome the capillary force of interfacial tension and encapsulate the bead.

The choice of suspension medium is also very important for the encapsulation and ejection process. $1\ cSt$ silicone oil offers the following advantageous with respect to the operation of DBDR:

1. It is insoluble in water or aqueous reagents. This makes DBDR compatible with a broad range of aqueous synthesis reactions.
2. As is shown in Fig. 3c of main text, in a suspension medium of higher viscosity, a higher fraction of the kinetic energy of the droplet motion is lost in viscous damping. Therefore, the droplet cannot move further away from the electrode to completely detach from the bead. The low viscosity of $1\ cSt$ silicone oil is advantageous in this regard.
3. Silicone oil is chemically inert. It does not undergo any unwanted chemical interactions with the initiator strands on the bead or with the reagents within the droplet.
4. It has a high dielectric breakdown strength of $13.8\ V/\mu m$[S10] which is higher than the maximum electric field of $1.33\ V/\mu m$ we can apply between the trap electrode and the ground. This avoids breakdown of the suspension medium under the entire range of operating conditions which otherwise can lead to shorting of electrodes. The resultant large short-circuit current between the electrodes will heat up and potentially boil off the fluid in the device. It may result in electric sparks within the device as well. The high breakdown strength of the silicone oil avoids all such scenarios.
5. The low electrical conductivity ($\sigma_0 = \sigma_{medium} = 10^{-14} S/m$) of silicone oil minimizes joule heating[S11,S12] ($\propto \sigma_0 |E|^2$) of the suspension medium due to the electric field between the trap electrode and the ground pad. This also avoids nonuniform heating of the suspension medium due to the spatially varying electric field that could result in

significant electrothermal fluid flow. The resultant drag force may hamper the encapsulation and ejection process.

6. The absence of ions in the silicone oil avoids electroosmotic[S12] fluid flow.

Factors 5 and 6 are critical to ensure the dominant electric field effect is the dielectrophoretic force.

The surfactant Span80 is also critical to the implementation of the encapsulation and ejection of the bead from the device. It reduces the interfacial tension between the silicone oil and the reagent and increases the contact angle that the droplet forms on the surface of the bead to $145°$. So, the combination of 1 cSt silicone oil and Span80 surfactant is essential to make the hydrophilic streptavidin coated polystyrene beads (reagent forms a contact angle of $45°$ on a streptavidin surface in air.) hydrophobic. This enables the ejection of the bead from the droplet on reducing the supply voltage (Fig. 4d of main text).

Supplementary Note 5: Particle tracking simulation of bead motion in synthesis columns on introduction of reagents.

These simulations were carried out to explain that the ideal case of perfect bead-bead stacking (which represents the case of minimum exposure of solid support bound growing strands of oligonucleotides to the influx of synthesis reagents) is seldom achieved in synthesis columns. To this end, we implement particle tracking simulations to show the displacement of beads from perfectly stacked initial positions due to turbulent motion of the reagent fluid as they are injected into the synthesis columns. As the beads move away from a perfectly stacked configuration, they have higher access to reagents which decreases again as the beads eventually settle down after the reagent injection stops into a stacked configuration. So, overall, the reaction fidelity and the fluorescence intensity on the beads lies between the lower limit of a perfectly stacked configuration and the upper limit of maximum exposure as in the case of DBDR.

For simplicity of setup and reduced memory and simulation time requirements 2D mirror symmetric simulations were set up in COMSOL Multiphysics (Supplementary Figure 12). As a further simplification, instead of a completely coupled turbulent flow-particle tracking simulations, the steady state solution of the turbulent flow equations was obtained using the Reynolds-averaged Navier-Stokes (RANS) model for turbulence[S13]. This steady state solution of the turbulent flow equations was used to exert drag force[S1, S14] on the particles (eq. S24) in the time dependent particle tracking simulations. The particles also experienced the force of gravity (eq. S25). The fluctuating components of the turbulence velocities are represented using a continuous random walk model. Assuming that the solutions in the cylindrical columns would be azimuthally symmetric, the solutions of the particle tracking simulations were revolved around the symmetry axis (Supplementary Figure 12) to generate the plots in Fig. 5 of main text.

Once the fluid flow subsides, the particles settle down under the force of gravity into a stack[S1,S14].

$$\vec{F}_{drag} = \frac{m_p}{\tau_p}\left(\vec{u}' - \vec{v}_p\right) \quad \text{(eq. S24)}$$

$$\vec{F}_{gravity} = \frac{\rho_p - \rho_f}{\rho_p} m_p \vec{g} \quad \text{(eq. S25)}$$

Here m_p is the mass of a particle, $\rho_p = 1050\ kg/m^3$ is its density, $\rho_f = 1000\ kg/m^3$ is the density of the fluidic suspension medium, $\vec{g} = 9.8\ kg/m^2$ is the acceleration due to gravity, $\tau_p = \frac{\rho_p d_p^2}{18\mu}$, $\vec{v}_p$ is the velocity of a particle and $\vec{u}' = \vec{u} + \vec{u}_f$ is the velocity of the fluid flow which is the sum of the mean flow velocity ($\vec{u}$) and the fluctuation term ($\vec{u}_f$).

Supplementary Note 6: Electric field driven enhancement of reagent concentration within droplet

The mobile reagents within the microdroplet consist of the negatively charged nucleotides, the enzyme (TdT) and the positively charged ions (Co^{2+} and Mg^{2+}) which are essential ingredients for optimal enzymatic activity. The A.C. electric field used to generate the dielectrophoretic force will also drive the migration of these ions leading to the accumulation of positively/negatively charged reagent species on the surface of the droplet closer to the bead during the opposing phases of the A.C. supply cycle (Supplementary Figure 14). Here we describe the details of the numerical simulation used to analyze the electric field driven migration of the ionic reacting species:

The reagent consisted of a mixture of component A ($25\,\mu l$) and component B ($3.25\,\mu l$). Component A consists of $19.55\,\mu l$ of deionized water, $2.65\,\mu l$ of $2.5\,mM\ CoCl_2$, and $2.65\,\mu l$ of $2.5\,mM$ buffer. The buffer which has a pH of 7.9 at 25℃ has $50\,mM$ potassium acetate, $20\,mM$ Tris-acetate, and $10\,mM$ magnesium acetate. It also has $5\,\mu M$ of fluorescently labelled nucleotides ($dCTP - AF647$). The component B is the TdT storage buffer which consists of $50\,mM\ K_3PO_4$, $100\,mM\ NaCl$, $1.43\,mM$ $2-$mercaptoethanol, 0.1% Triton-X-100 and 50% glycerol. The TdT storage buffer has $20U/\mu l$ of TdT. The concentration (c_i) of the various ions in this reagent mixture along with their diffusion coefficient (D_i) and charge (z_i) is outlined in Supplementary Table 4.

The A.C. electric field driven modulation in the ion concentration is governed by the following equations[S1,S14]:

$$\frac{\partial c_i}{\partial t} = \vec{\nabla} \cdot \left(-D_i \vec{\nabla} c_i - z_i \mu_i F c_i \vec{\nabla} V\right) \quad \text{(eq. S27a)}$$

$$\mu_i = \frac{D_i}{RT} \quad \text{(eq. S27b)}$$

Here, μ_i is the mobility of ions, F is the well-known Faraday constant, and V is the electrical potential distribution which is evaluated using simulations outlined in Supplementary Figure 14, R is the universal gas constant, T is the temperature, and i is the any one of the ionic species in Supplementary Table 4. These equations are solved using COMSOL Multiphysics®. The concentration enhancement (ce_i) of any species is evaluated by averaging the normalized concentration ($\frac{c_i}{c_{io}}$) of the species over the surface of the bead. Here, c_{i0} is the concentration in the absence of the electric field. As can be seen during opposite phases of the A.C. supply cycle ($f_s = 200\,Hz$) the concentration of Mg^{2+}, Co^{2+}, and $Nucleotide^{3-}$ ($dCTP$ has charge -4 and the AF647 has charge $+1$) increases at the bead without the addition of more molecules into the reagent droplet (Supplementary Figure 15). The average concentration enhancement ($\langle ce_i \rangle$) of any charged species over one A.C. supply cycle in the steady state regime is evaluated using the following equation:

$$\langle ce_i \rangle = \frac{\int_0^T ce_i dt}{T} \qquad \text{(eq. S28)}$$

$\langle ce_{Co} \rangle = 0.91$ and $\langle ce_{dCTP-AF647} \rangle = 1.58$. High frequency A.C. cycles lead to lesser enhancement of concentration as the ion migration cannot keep pace with the faster switching of voltages (Supplementary Figure 16). On the other hand, although D.C. supply will provide maximum ion concentration, it will enhance the concentration of only a single charge (positive or negative). Low frequency $A.C.$ helps achieve higher concentration enhancement ($\langle ce_i \rangle$) of both positively and negatively charged reacting species at the surface of the bead during opposing halves of the AC supply cycle. Larger supply voltage also increases $\langle ce_i \rangle$ (Supplementary Figure 17).

The increased interaction of charged reacting species with the solid support may also contribute to the improved reaction fidelity in DBDR compared to traditional synthesis columns.

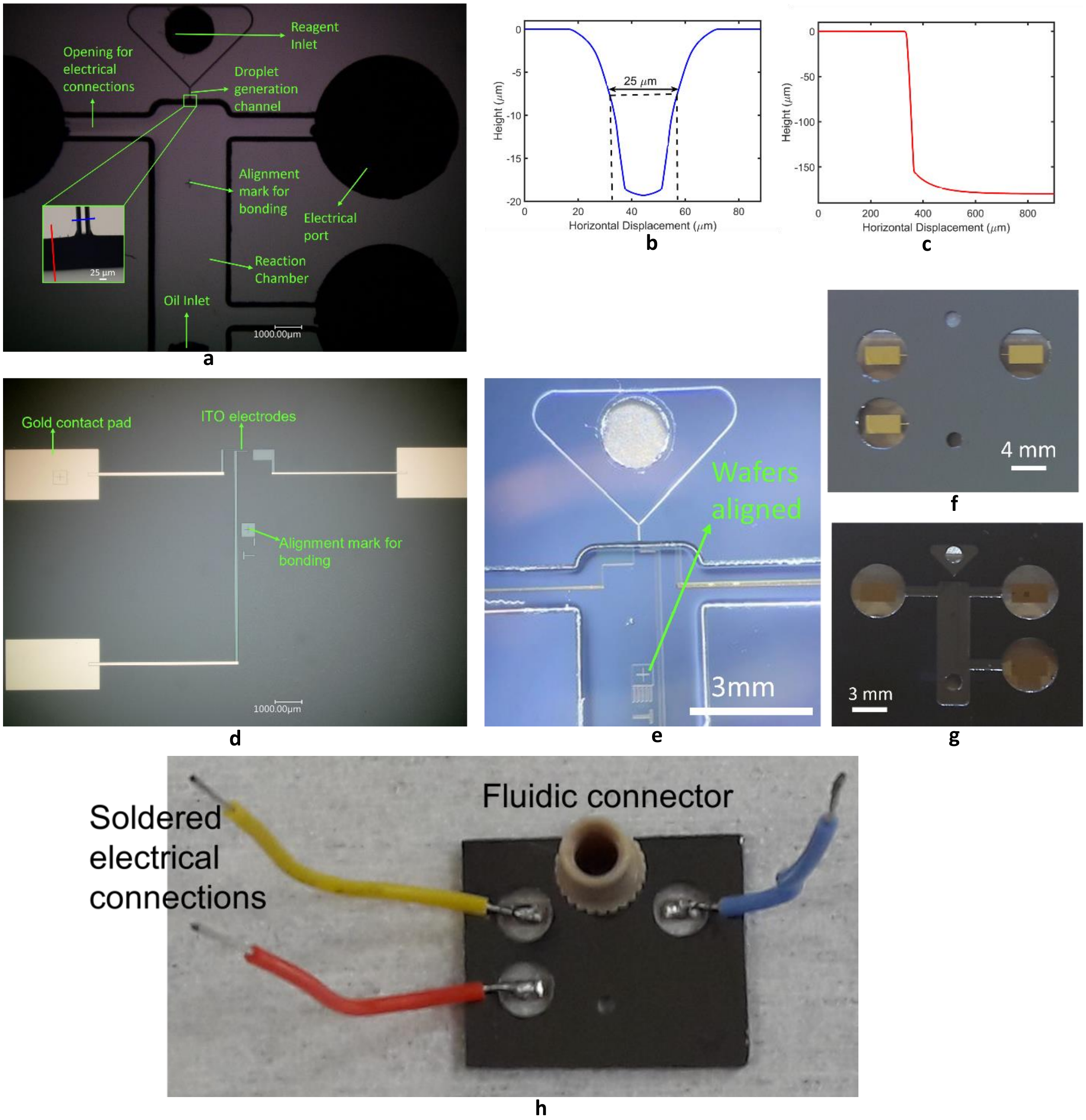

Supplementary Figure 1. Device Fabrication. (a) Fabricated microfluidic components on silicon. The inset gives a magnified image of the channel opening into the reaction chamber. (b) and (c), represent the spatial depth profile along the blue and red lines which give the depth of the channel and the reaction chamber. (d), fabricated electrical components on the glass. (e), The silicon and the glass part of the device are aligned using the alignment markers in an EV Aligner system. They are then bonded and diced into chips. (f**)**, The top (port) view and the (g), bottom (device) view of the fabricated silicon-on-glass microfluidic device. (h), External electrical and fluidic connectors attached to the device ports.

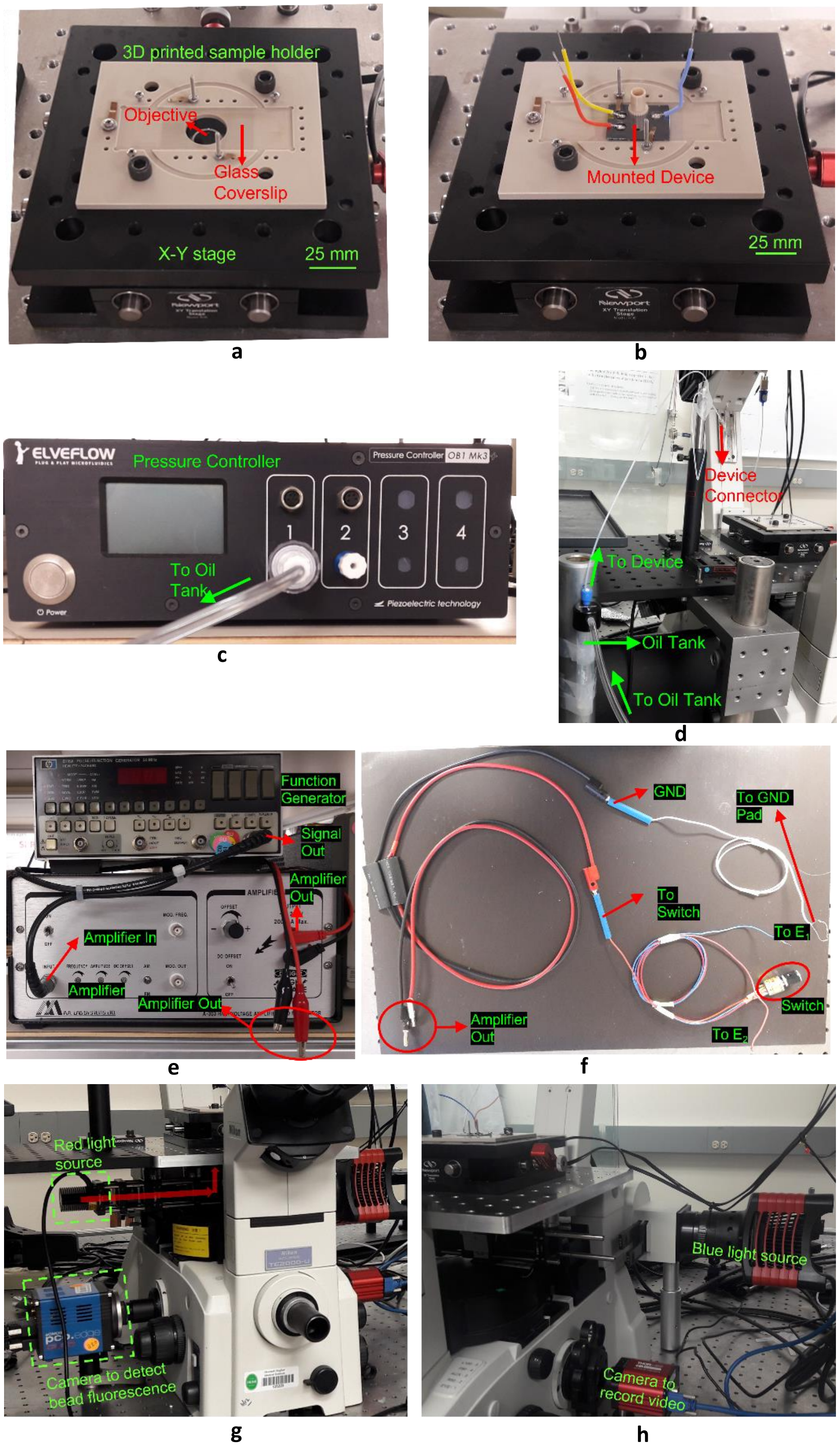

Supplementary Figure 2. Experimental Setup (a) The 3-D printed sample holder is screwed onto the x-y stage which sits on an inverted microscope setup. (b) The device is mounted on the sample holder on top of a glass coverslip to prevent any fluid flow onto the objective. (c) The piezo-driven pressure controller (OB1 MK3+) from Elveflow drives fluid flow through the microfluidic device. The output of the pressure controller is connected to the oil tank. (d) The pressurized air from the pressure controller drives

fluid flow from the oil tank into the device. The device connector connects the output from the oil tank to the device. (e) The electrical signal (up to 15V amplitude) from the function generator is fed to the amplifier which amplifies the signal by 20x. (f) The amplified output of the signal generator is supplied to either of the electrodes (E_1 or E_2) using a single pole double throw switch. (g) The red-light source is used for the fluorescent detection of the labelled nucleotide. (h) The blue light source is used for imaging fluid flow, the fluorescent green beads, and the encapsulation and ejection of the bead by the droplet.

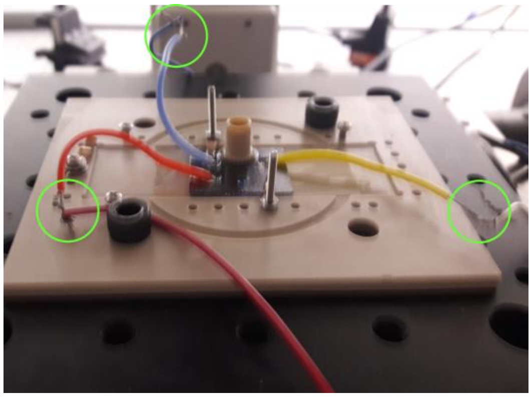

a

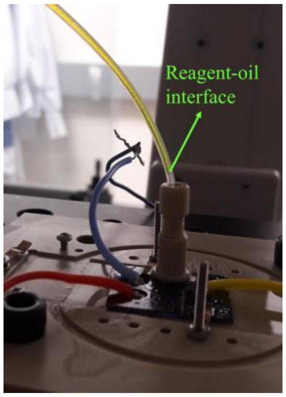

b

Supplementary Figure 3. Sample Mounting (a) The device filled with oil is mounted on the sample stage and electrical connections made to the three device terminals as shown within the three green circles. (b) Fluidic connection made to the device using the N-333 connectors. The bottom of the tubing is filled with oil to ensure the reagent does not flow into the device as soon as the connection is established for better experimental control. The reagent flows in after the oil. The reagent-oil interface in the input tubing is shown.

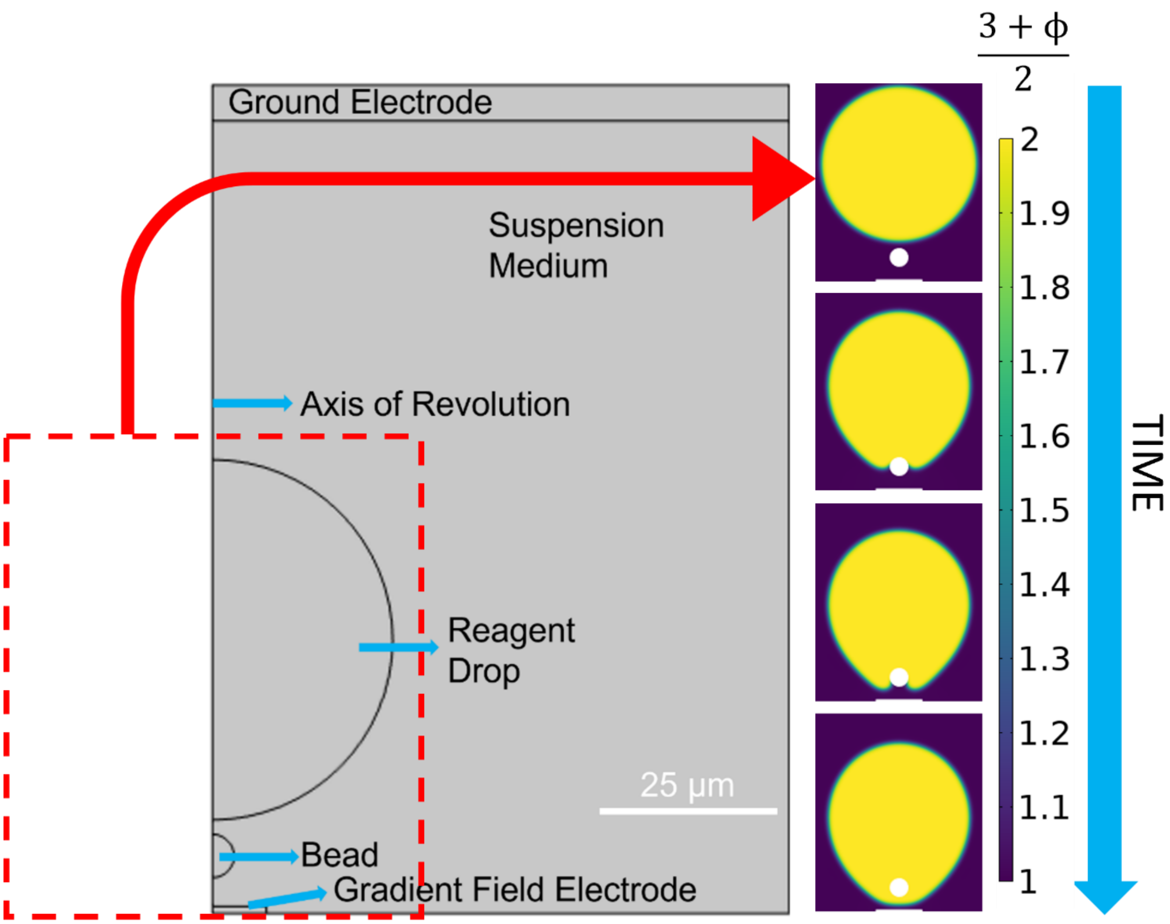

Supplementary Figure 4. Electrohydrodynamic simulation setup. Axis-symmetric simulation structure used for electrohydrodynamic simulations in COMSOL Multiphysics ®. Two-dimensional simulation time frames are taken from the simulation setup by defining a cut plane (red dashed region).

Variable	Value	Description	Reference
R_b	3 µm	Radius of bead	6µm diameter streptavidin coated green, fluorescent polystyrene beads purchased from polysciences (Catalog No.#-24157).
R_d	25 µm	Radius of drop	Estimated from measurements in Supplementary Information Fig. S1c
R_{grad}	15 µm	Radius of gradient field electrode	From designed dimension of the electrode Fig. 1(b)
R_{ground}	80 µm	Radius of ground electrode	Chosen to make the simulation space sufficiently larger than the dimensions of the bead and droplet under consideration.
R_{medium}	80 um (=R_{ground})	Radius of suspension medium	Same as R_{ground} due to the system geometry
h_{grad}	1 µm	Thickness of gradient field electrode	Approximate thickness of evaporatively deposited Indium Tin Oxide electrode which is 0.8µm.
h_{ground}	5 µm	Thickness of ground electrode	This choice does not affect the simulation in any way.
h_{medium}	115 µm	Thickness of medium	Spacing between the electrode and ground in Fig. 1(b)
ρ_{drop}	1025 kg/m^3	Density of reagent phase	Evaluated by considering the density of all the ingredients of the reaction buffer.
ρ_{SO}	818 kg/m^3	Density of oil	Density of silicone oil (CAS#-105-51-7)
ρ_{Span80}	986 kg/m^3	Density of Span80	Density of Span80 (CAS#-1338-43-8)
V_{oil}	200 ml	Volume of silicone oil	
V_{Span80}	4 ml	Volume of Span80	
V_{med}	V_{oil} + V_{Span80}	Volume of medium	Approximate volume of oil + Span80
V_{of}	200/204	Volume fraction of oil	V_{oil}/V_{med}
$V_{Span80f}$	4/204	Volume fraction of Span80	$V_{Span80f}/V_{med}$
ρ_{medium}	821 kg/m^3	Density of medium	$\rho_{SO}\ V_{of}+\ \rho_{Span80}\ V_{Span80f}$
η_{drop}	8.9e-4 Pa.s	Dynamic viscosity of reagent drop	Assumed to be dynamic viscosity of water
η_{SO}	8.18e-4 Pa.s	Dynamic viscosity of 1cSt silicone oil	$= \kappa_{SO} \times \rho_{SO}$ (κ_{SO} is the kinematic viscosity which is 1 cSt)
η_{Span80}	1 Pa.s	Dynamic Viscosity of Span80	Viscosity of Span80 (CAS#-1338-43-8)

η_{medium}	1.5e-3 Pa.s	Dynamic viscosity of suspension medium	$\eta_{medium}^{1/3} = x_{SO}\eta_{SO}^{1/3} + x_{Span80}\eta_{Span80}^{1/3}$ [S15]
ε_{drop}	80	Relative permittivity of drop	Relative permittivity of water
ε_{medium}	2.30	Relative permittivity of medium	Relative permittivity of silicone oil (CAS#-105-51-7).
ε_{bead}	2.55	Relative permittivity of bead	[S16]
σ_{drop}	6e-1 S/m	Electrical conductivity of reagent drops	Measured
σ_{medium}	1e-14 S/m	Electrical conductivity of suspension medium	[S17]
σ_{bead}	1e-9 S/m	Electrical conductivity of bead	Conductivity of polystyrene bead suspended in silicone oil [S18].
Γ	5.5 mN/m	Interfacial tension between the reagent droplet and the suspension medium	Measured. Fig. 3 main text
θ_{bead}	145°	Contact angle of the reagent drop on the surface of the streptavidin coated bead surrounded by the oil medium.	Estimated Fig. 3 main text
θ_{wall}	140°	Contact angle of the reagent drop on the device walls.	Estimated Supplementary Information Fig. S1d

Supplementary Table 1. Table of all the geometric and material parameters used in electrohydrodynamic simulations and energy calculations.

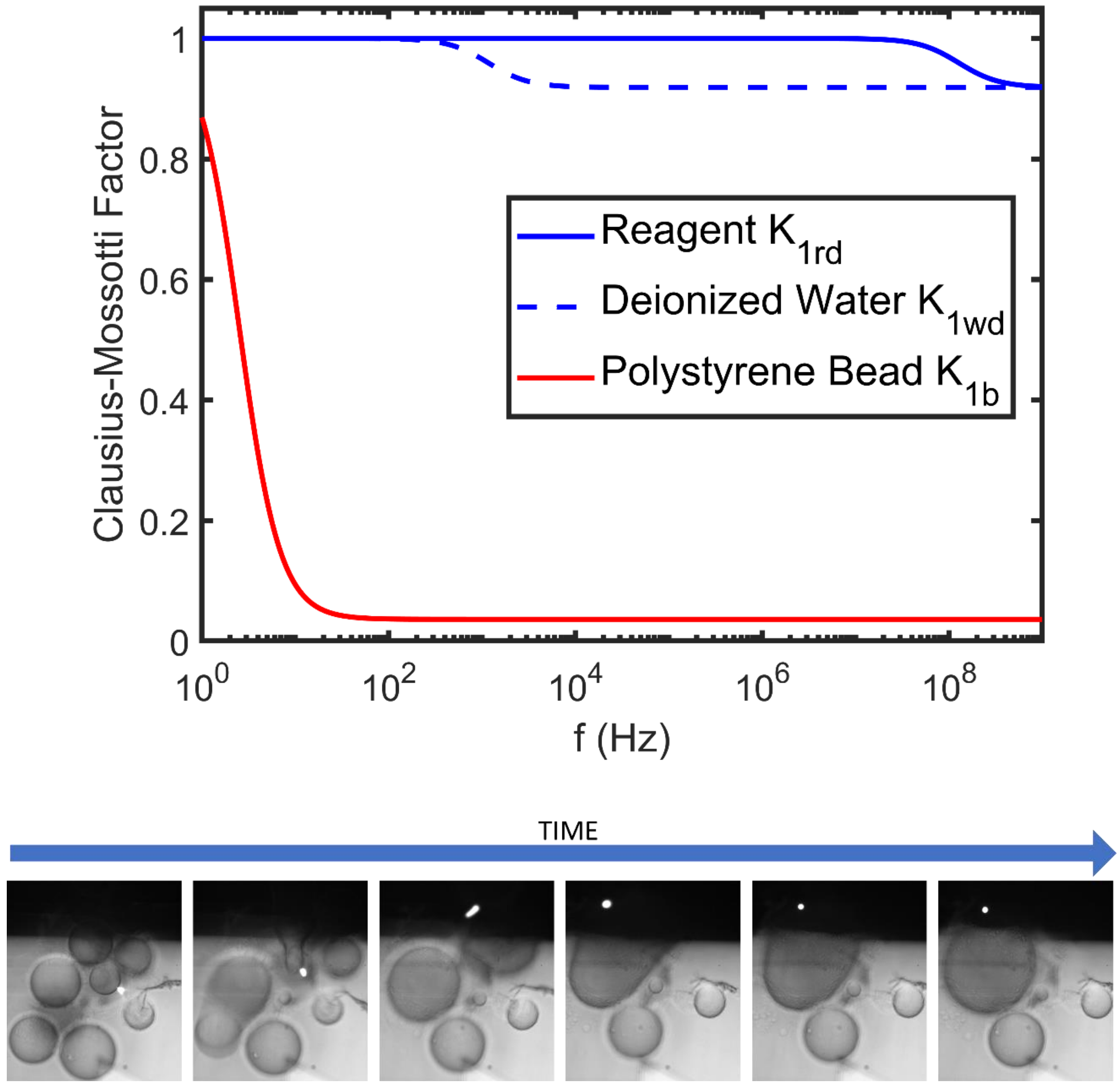

Supplementary Figure 5. Electric field response of beads and droplets. (a) Clausius-Mossotti factor for various particles suspended in silicone oil solution. K_{1rd}, K_{1wd}, and K_{1b} are the Clausius-Mossotti factor for reagent droplet, water droplet and polystyrene bead suspended in 1 cSt silicone oil with 2.5% w/w Span80 added to it. (b) The larger Classius-Mossotti factor of reagent droplets makes them more sensitive to the electric field. So, even at smaller voltages ($\approx 20\ V$) the larger dielelctrophoretic force on the droplets makes droplet-droplet merger the primary dielectrophoretic effect.

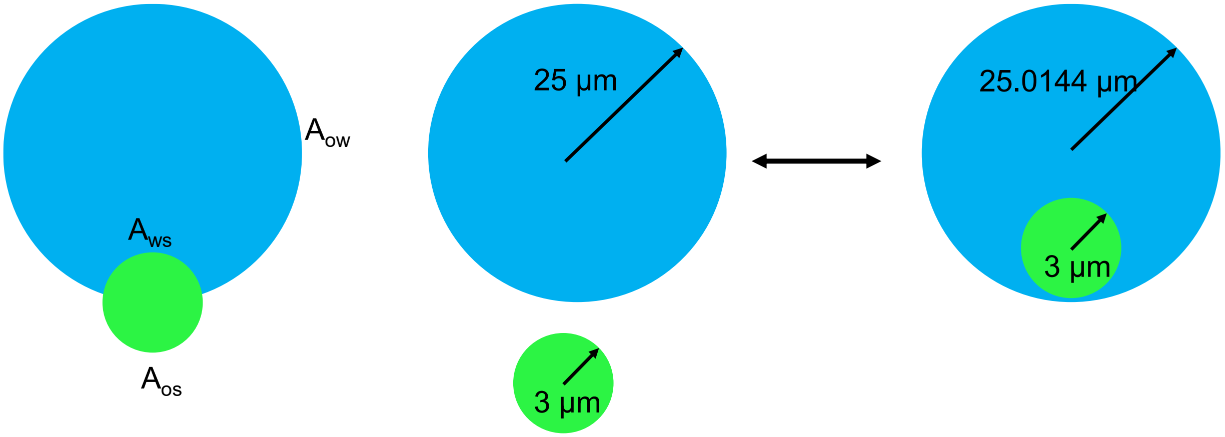

Supplementary Figure 6. Change in the droplet dimension with the encapsulation of the bead into the droplet. As the bead is encapsulated within the droplet, its radius (R_d) increases slightly. This leads to a slight increase in its surface area (A_{ow}) with the surrounding oil medium.

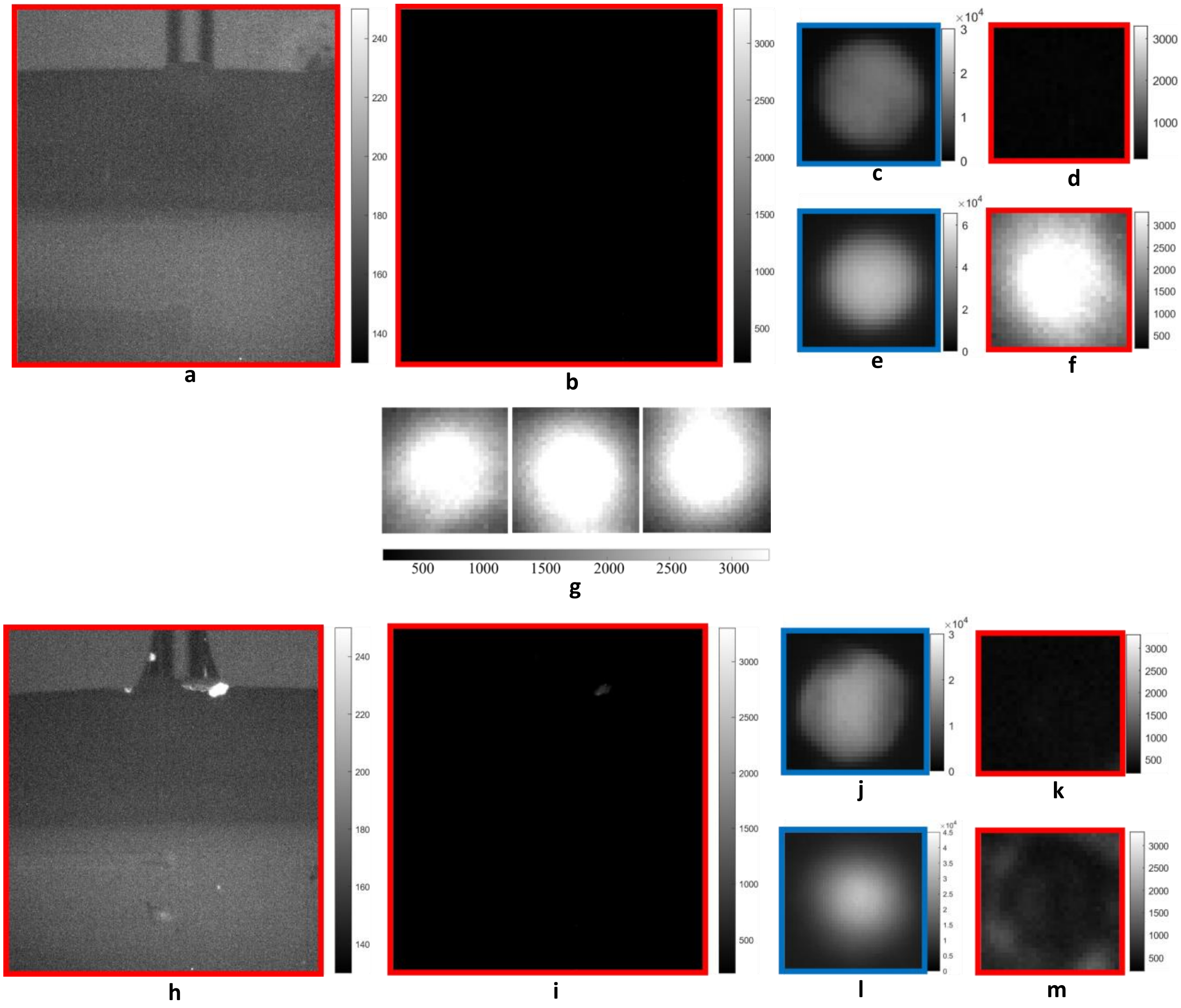

Supplementary Figure 7. Fluorescence images and intensity data for enzymatic coupling and control experiment. (a)-(f) Coupling reaction. (g) Red fluorescence images of beads from three consecutive reactions. (h)-(m) Control reaction. (a) and (h) Device filled with oil and with relevant electrical and fluidic connectors before start of experiment with color scale adjusted to lower thresholds and (b) and (i) higher thresholds. (a) and (b) ((h) and (i)), are the same image with different color scales. The lower color scale in (a) and (h) is used to highlight the device structure under red illumination while the higher color scale in (b) and (i) is used to depict the absence of any source of noise at the level of the fluorescence signal from the reacted beads due to AF-647 used to label dCTP. (c) and (j) Bead before encapsulation and ejection from droplet under blue and (d) and (k)**,** red illumination. (e) and (l), Bead after encapsulation and ejection from droplet under blue and (f) and (m), red illumination. The color of the boxes denotes the illumination source (blue $\lambda_{ex} - 455\ nm$, red $\lambda_{ex} - 637$ nm).

Reagent	Ml
TdT (20 U/ µl)	3.0
Buffer (10 x)	2.5
$CoCl_2$ (2.5 mM 10 x)	2.5
Beads (7 µl) or initiator (50 µM*)	0 (or 1)
Dye (1 mM)	0.1
Water*	16.9 (or 15.9)
**Total**	25

a

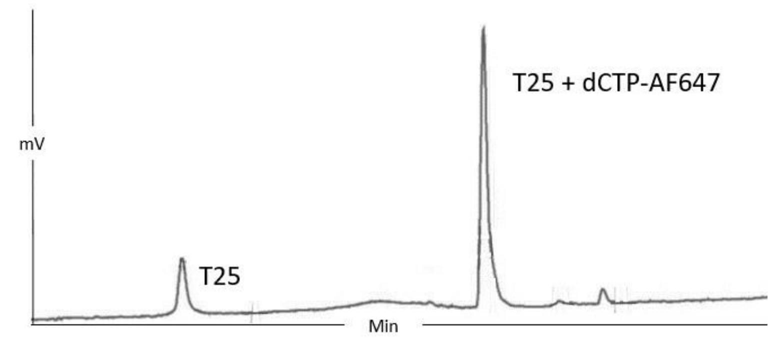

b

Supplementary Figure 8. Benchtop synthesis reaction. (a) Either the reaction was prepared 1) for synthesis free in-solution (no beads) for parameter optimization using high performance liquid chromatography (HPLC) where 1 µl (50 µM strand (T25) was added to the reaction mix or 2) for synthesis directly onto the beads (in this case 7 µl beads with the initiator already attached were used); for experiments performed in a column (Fig. 5 and 6 of main text), reaction volumes were doubled to 50 µl. In both cases (1 and 2), 10 M EDTA was used to stop the synthesis reaction after 5 min. Buffer contents: 50 mM potassium acetate, 20 mM Tris-acetate and 10 mM magnesium acetate. (b) HPLC chromatogram showing general results for post-synthesis on the benchtop (5 min, 23°C) free in-solution (T25mer coupled to dCTP-AF647); HPLC analysis was only performed to optimize synthesis conditions before translating to the device. Parameters for sample processing include, Hitachi WAV system, DNA-Sep column (C-18, Cat. DNA-99-3510); method conditions: Buffer A (0.1 M TEAA (triethylammonium acetate) in water), Buffer B (0.1 M TEAA (ADS Biotech), 25% acetonitrile (Sigma Aldrich)), 80°C, 1.2 ml/ min, gradient: 82% to 12% A for 10 min; absorbance and fluorescence were measured at 260 nm and 648 nm (excitation)/ 688 nm (emission), respectfully. Five µl directly aspirated from the reaction were added to the HPLC column.

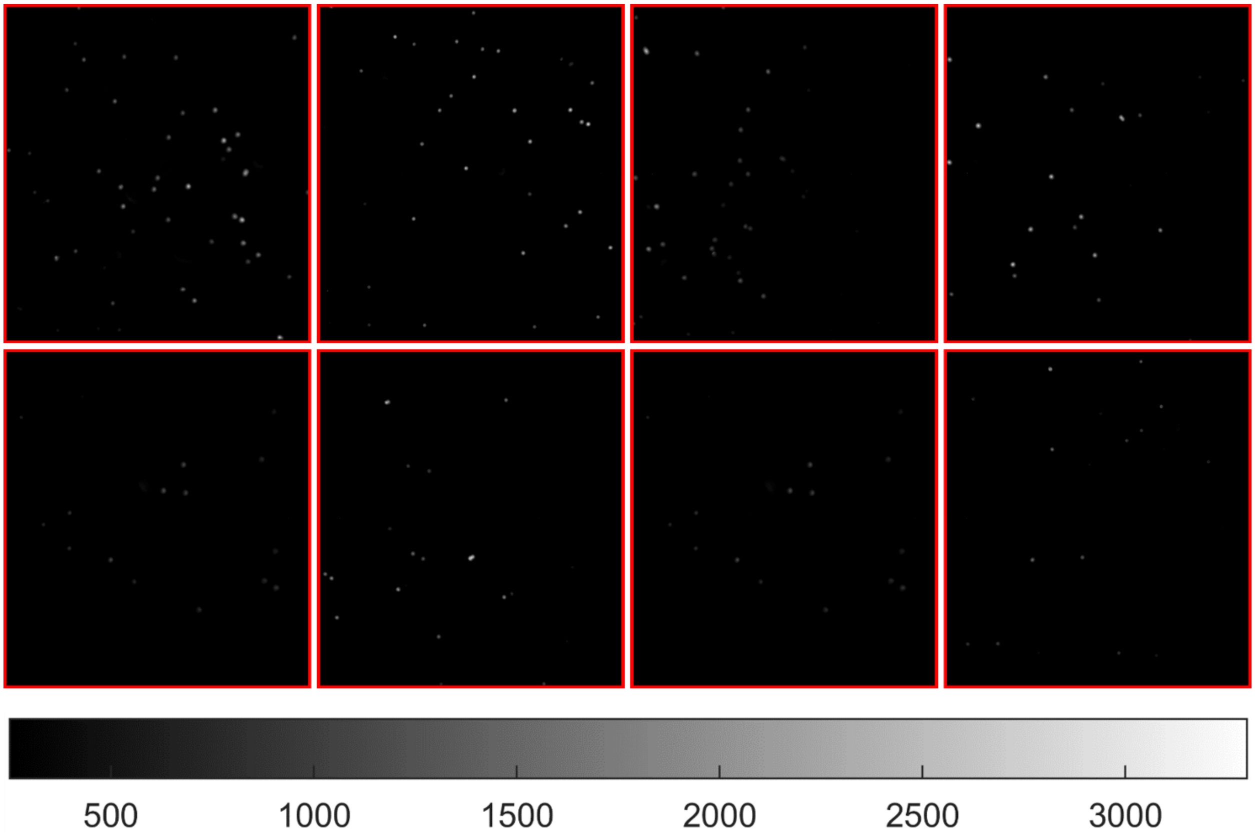

a

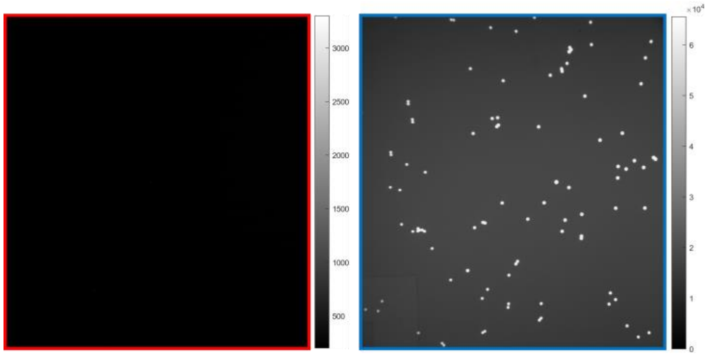

b

Supplementary Figure 9. Fluorescence data for coupling and control reactions implemented in synthesis columns (benchtop reactions). (a) Beads with enzymatic coupling of fluorescently labelled (AF647) bases show a large variation in fluorescent intensity which indicates a large variation in the degree of coupling. Images were captured as 16-bit Tiff files using PCO.EDGE 5.5 camera with an integration time of 2 s under red excitation ($\lambda_{ex} = 637\ nm$). A few representative snapshots are shown here. (b) Control experiment implemented on beads without initiator strands exposed to coupling reagents without initiator strands shows no red fluorescence under red excitation ($\lambda_{ex} = 637\ nm$). The beads fluoresce green under blue excitation. This is used to detect the physical presence of the beads within the field of view of the microscope. The outline color of the images indicates the excitation source (blue $\lambda_{ex} - 455\ nm$, red $\lambda_{ex} - 637$ nm).

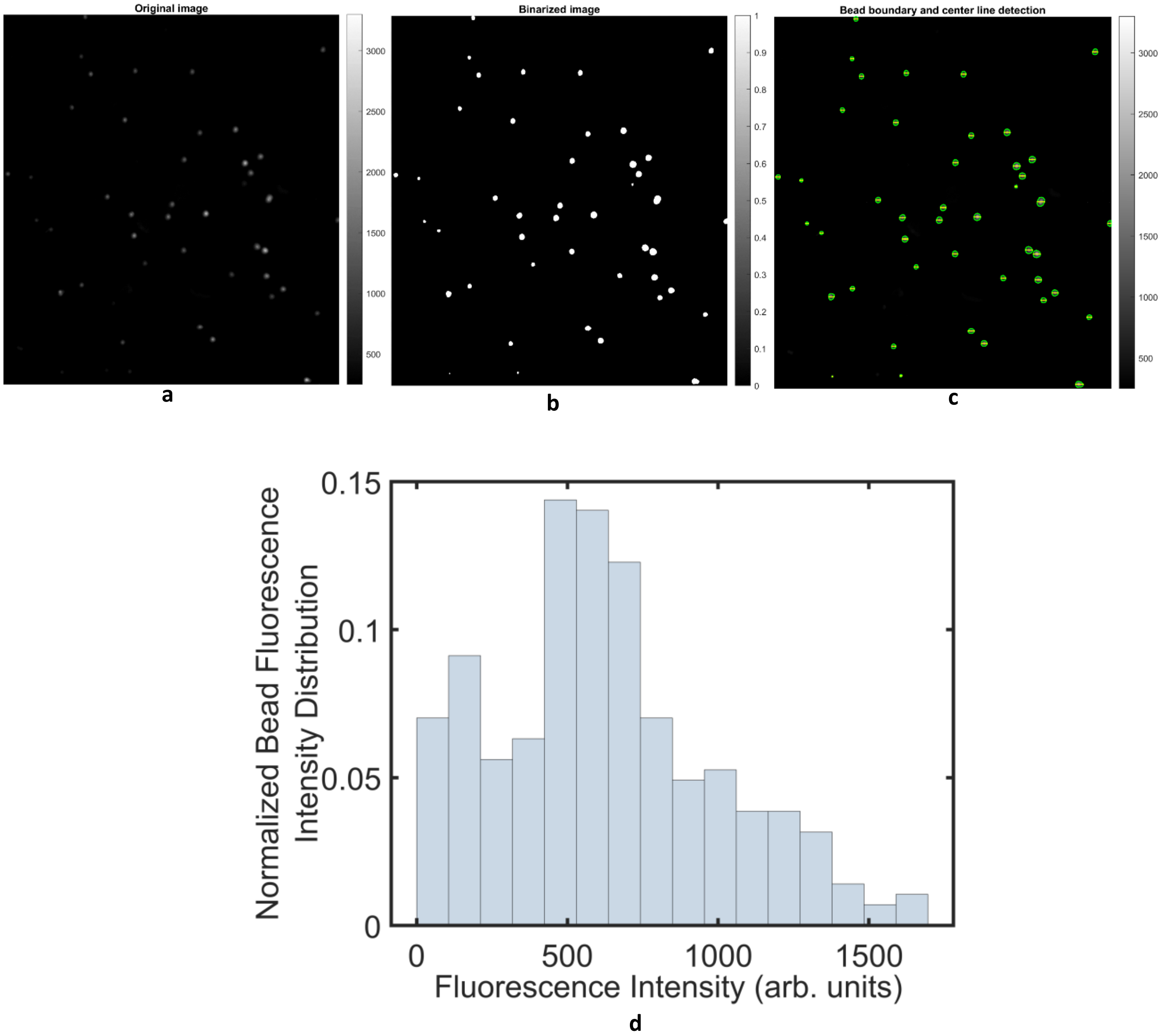

Supplementary Figure 10. Image processing of benchtop reaction snapshots to extract fluorescence data from beads. (a) A 16-bit snapshot of fluorescent beads post enzymatic coupling of fluorescently labelled nucleotides in benchtop setups under red excitation ($\lambda_{ex} = 637\ nm$). It is the same as the first snapshot in Fig. 6a. (b) A binary transformation of the image in (a). (c) Edge and center line detection of the binarized images in (b). The extracted fluorescence intensity patterns across the center lines are plotted in Fig. 4d of the main text. The transformations are applied to all the collected bead snapshots. (d) Average fluorescence intensity distribution of all the beads across all the frames.

Statistical Parameter	Synthesis Columns	DBDR
Number of samples (N)	285	3
Mean (μ)	627.6	2057.5
Standard Deviation (σ)	373	209.04

a

Hypothesis Testing Parameter	Value
t	11.65
df	2.14
α	0.05
t^*	4.30
power	1

b

Supplementary Table 2. Statistical significance testing for reaction fidelity enhancement using Welch t-test on fluorescence intensity data. (a) Statistical parameters obtained from experimental data. (b) Evaluated parameters for Welch t-test.

Statistical Parameter	Synthesis Columns	DBDR
Number of samples (N)	285	3
Mean (μ)	143	287
Standard Deviation (σ)	82.27	0.82

a

Hypothesis Testing Parameter	Value
t	29.41
df	285.79
α	0.05
t^*	1.9683
power	0.97

b

Supplementary Table 3. Statistical significance testing for reaction fidelity enhancement using Welch t-test on ranks of fluorescence intensity data. (a) Statistical parameters obtained from rank transformation of experimental data. (b) Evaluated parameters for Welch t-test.

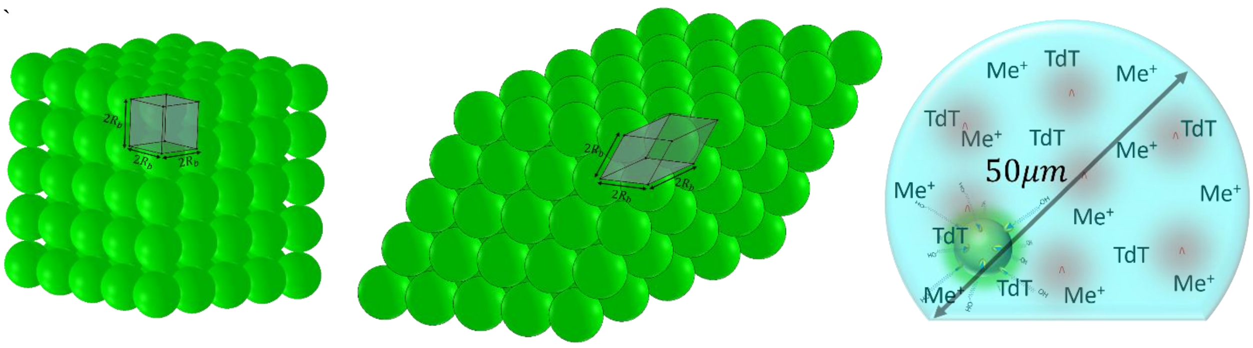

(a)

	Simple Cubic	Rhombohedral	Bead-Droplet Reactor
Number of initiators on bead surface	$100\ attomoles$	$100\ attomoles$	$100\ attomoles$
Concentration of nucleotide in reagent	$5\ \mu M$	$5\ \mu M$	$5\ \mu M$
Reagent Volume/Void	$4\left(2-\frac{\pi}{3}\right)R_b^3 \approx 103fl$	$4\left(\sqrt{2}-\frac{\pi}{3}\right)R_b^3 \approx 40fl$	$\frac{2-3\cos\varphi+\cos^3\varphi}{3} \approx 65pl$
Number of nucleotides within reagent volume	$0.52\ attomoles \approx \frac{100}{192}\ attomoles$	$0.2\ attomoles \approx \frac{100}{500}\ attomoles$	$325\ attomoles \approx \frac{100}{0.3077}\ attomoles$

(b)

Supplementary Figure 11. Reagent access to bead surfaces in various packing configurations and in DBDR. (a) The simple cubic (on the left) represents one of the most loosely packed lattice arrangement of beads whereas the rhombohedral (in the center) is one of the most tightly packed arrangement. Our proposed dielectrophoretic bead-droplet reactor (DBDR) system consisting of a single bead in a droplet reactor. (b) Quantification of reagent access to the bead surfaces.

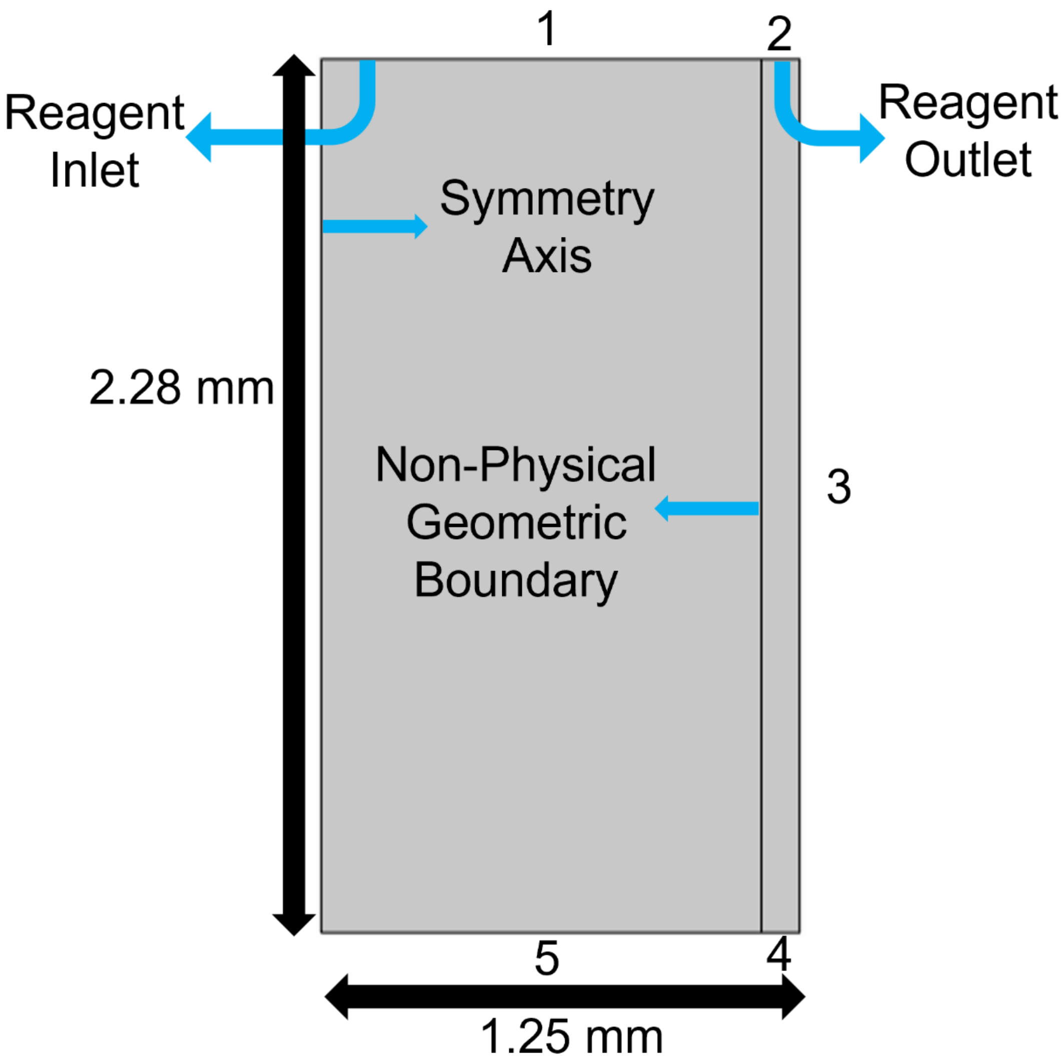

Supplementary Figure 12. Turbulent flow driven particle tracking simulation setup. Two-dimensional turbulent flow driven particle tracking simulation implemented in COMSOL Multiphysics® to study displacement patterns of particles from stacked configuration in synthesis columns as the reagent fluid is injected into the reaction space. 3-5 are walls for the fluid flow. 1-5 are walls for the particles as they are held in the space by filters. Reagent fluid enters from the top and tends to leak from the sides when the reaction space gets filled up. The filter at the bottom creates a resistive path for fluid flow vertically down, thus leading to its leakage from the sides.

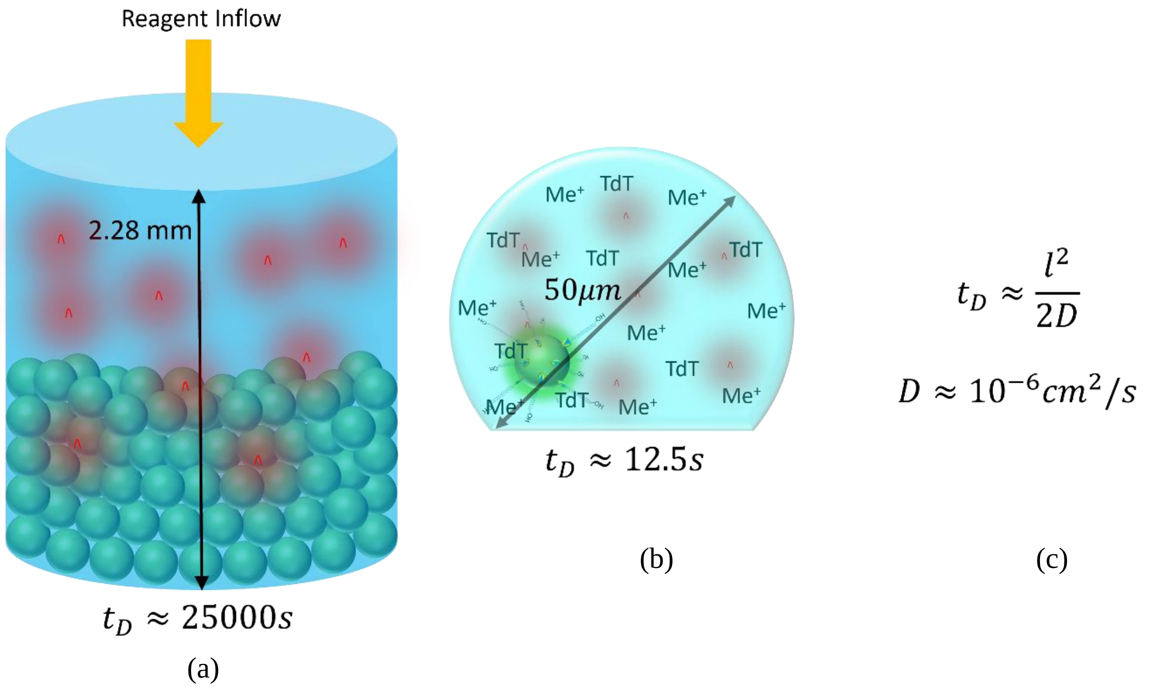

Supplementary Figure 13. Role of diffusion in reagent access to bead surfaces in stacked bead configurations and in DBDR. (a) Typical synthesis columns consisting of many beads stacked together are millimeters in dimensions. (b) Our proposed dielectrophoretic bead-droplet reactor (DBDR) system consisting of a single bead in a micron sized droplet reactor. (c) Mathematical formulation for the estimation of diffusion times.

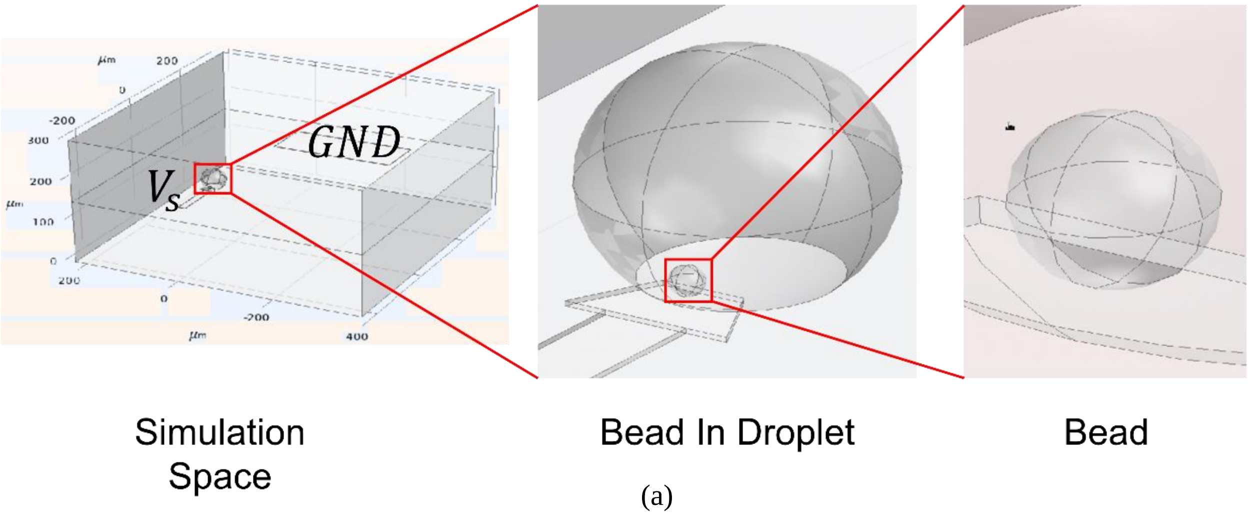

(a)

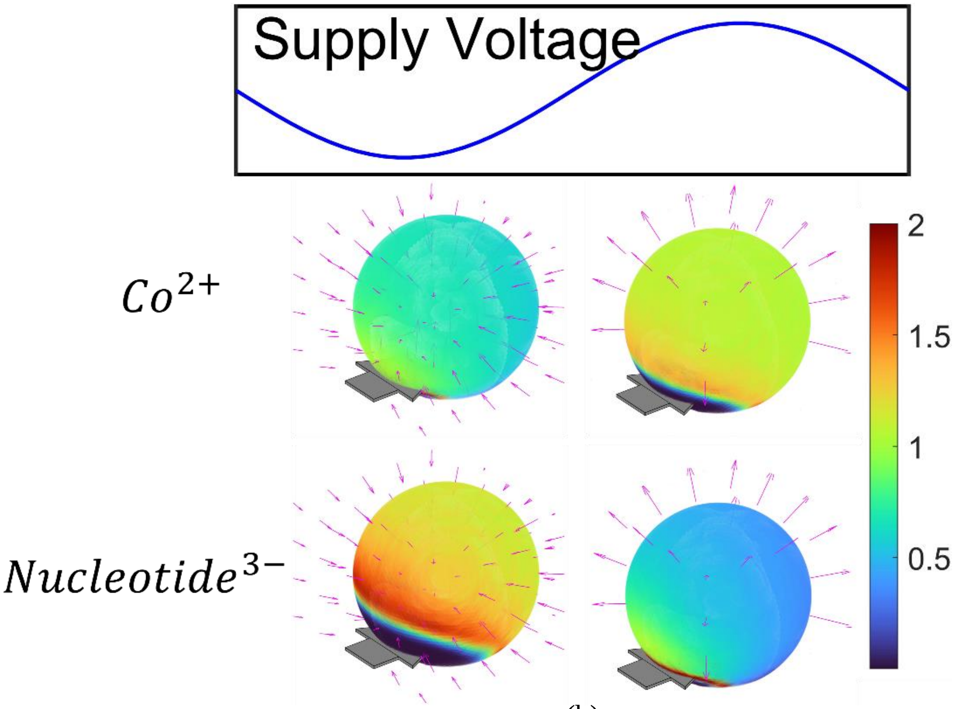

(b)

Supplementary Figure 14. Simulation for concentration enhancement of ionic species on the bead surface. (a) A bead is encapsulated within a droplet trapped on an electrode. An external A.C. voltage supply is connected across the electrode and the ground. (b) This leads to the accumulation of positive/negative charges on the surface of the droplet close to the trap electrode (and the solid support) during opposing phases of the A.C. supply cycle.

Species (i)	Concentration (c_i) in mol/m^3	Charge (z_i)	Diffusion Coefficient (D_i) in m^2/s
K (from potassium acetate and potassium phosphate)	14.8936	+1	$1.96e^{-9}$
Co	0.266	+2	$0.732e^{-9}$
Mg	1.0638	+2	$0.705e^{-9}$
Na	6.3830	+1	$1.33e^{-9}$
Cl (from cobalt chloride and sodium chloride)	6.9149	-1	$2.03e^{-9}$
CH_3COO (from potassium acetate and magnesium acetate)	7.4468	-1	$1.089e^{-9}$
Nucleotide	$5e^{-3}$	-3	$1e^{-9}$
PO_4	3.1915	-3	$0.612e^{-9}$

Supplementary Table 4. Summary of parameters used in electric field driven ion transport modelling.

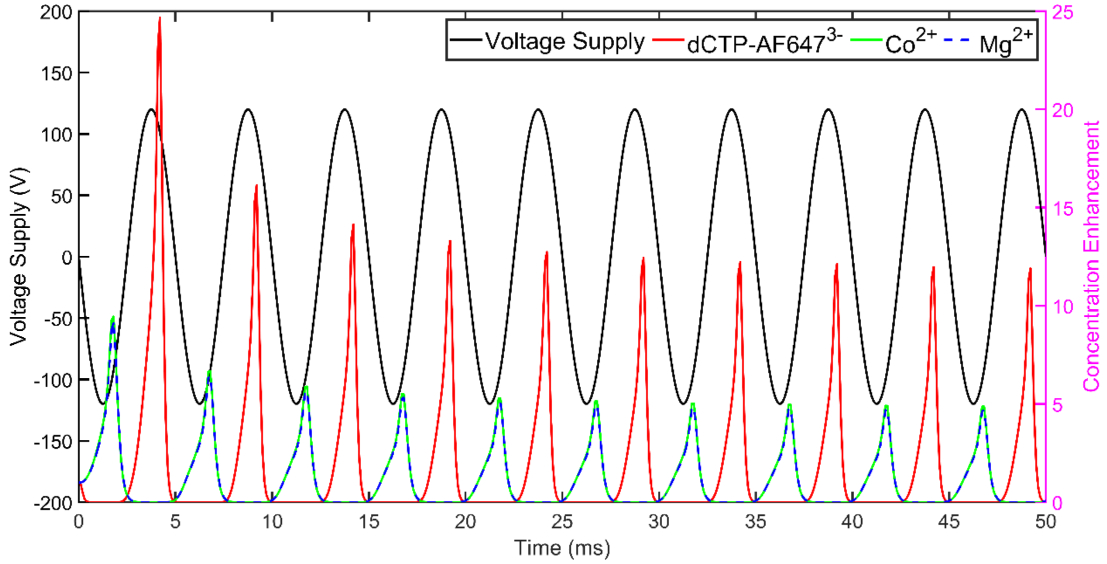

Supplementary Figure 15. Concentration enhancement of ionic reacting species during the A.C. supply cycle. The concentration of the positively and negatively charged species are enhanced during opposite phases of the A.C. supply cycle.

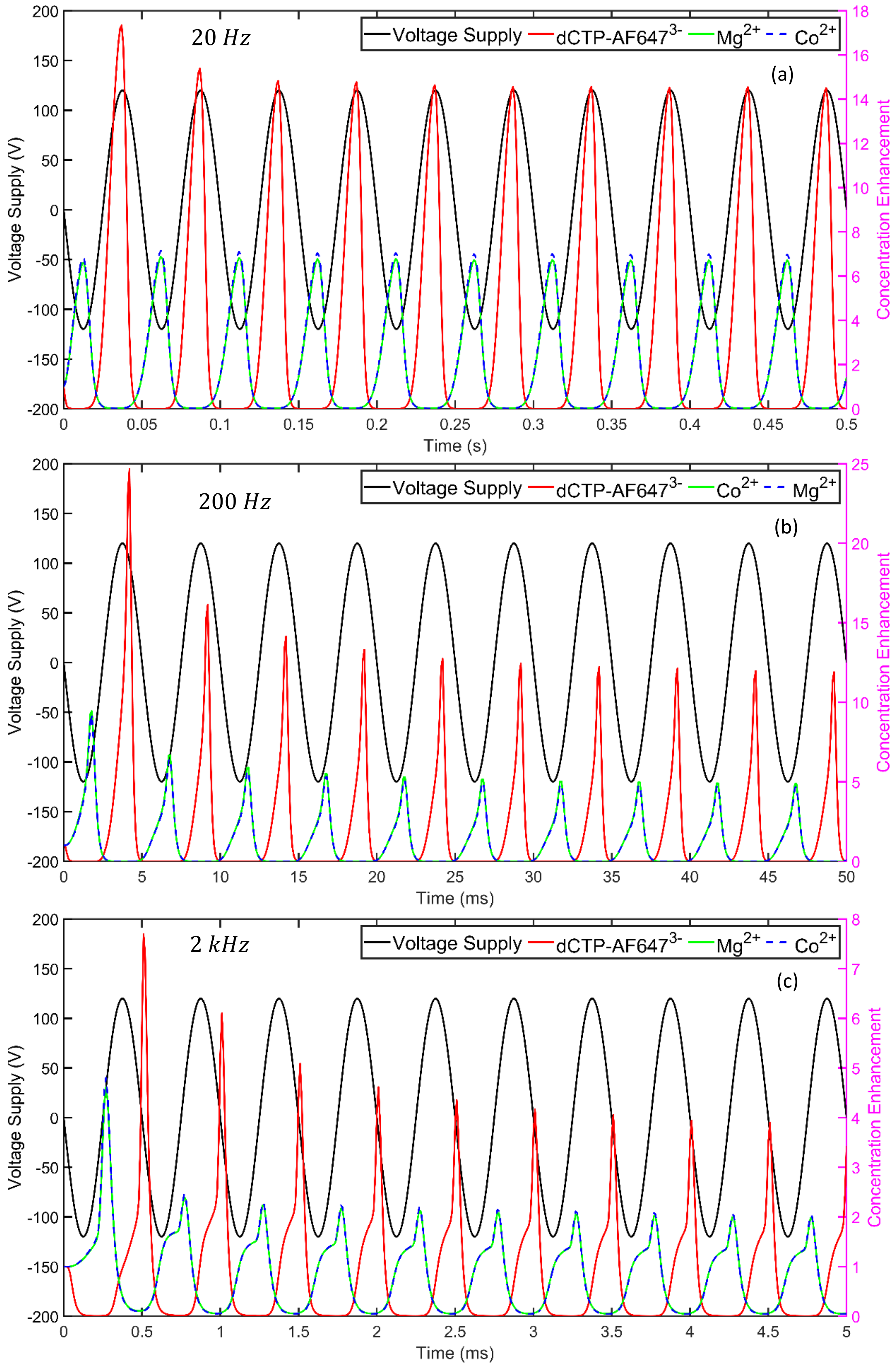
20 Hz
Voltage Supply
dCTP-AF647^{3-}
Mg^{2+}
Co^{2+}
(a)
Voltage Supply (V)
Concentration Enhancement
Time (s)
200 Hz
Voltage Supply
dCTP-AF647^{3-}
Co^{2+}
Mg^{2+}
(b)
Voltage Supply (V)
Concentration Enhancement
Time (ms)
2 kHz
Voltage Supply
dCTP-AF647^{3-}
Mg^{2+}
Co^{2+}
(c)
Voltage Supply (V)
Concentration Enhancement
Time (ms)

Supplementary Figure 16. Concentration enhancement of ionic reacting species during the A.C. supply cycle with varying supply frequency. (a) 20 Hz (b) 200 Hz and (c) 2 kHz. The ions can track the A.C. supply at smaller frequencies as in (a). Therefore, the ionic concentration enhancement closely represents the absolute value of a sine wave, and the mean values are higher ($\langle ce_{dCTP-AF647} \rangle = 3.39$, $\langle ce_{Co} \rangle = 1.86$, $\langle ce_{Mg} \rangle = 1.81$). As the A.C. supply frequency increases, the ions cannot track the fast-switching A.C. Supply. Therefore, the enhancements (at 200 Hz, $\langle ce_{dCTP-AF647} \rangle = 1.59$, $\langle ce_{Co} \rangle = 0.91$, $\langle ce_{Mg} \rangle = 0.89$, at 2 kHz, $\langle ce_{dCTP-AF647} \rangle = 0.64$, $\langle ce_{Co} \rangle = 0.68$,$\langle ce_{Mg} \rangle = 0.69$) are correspondingly lower and does not represent the absolute value of a sine wave. The voltage supply amplitude was 120 V.

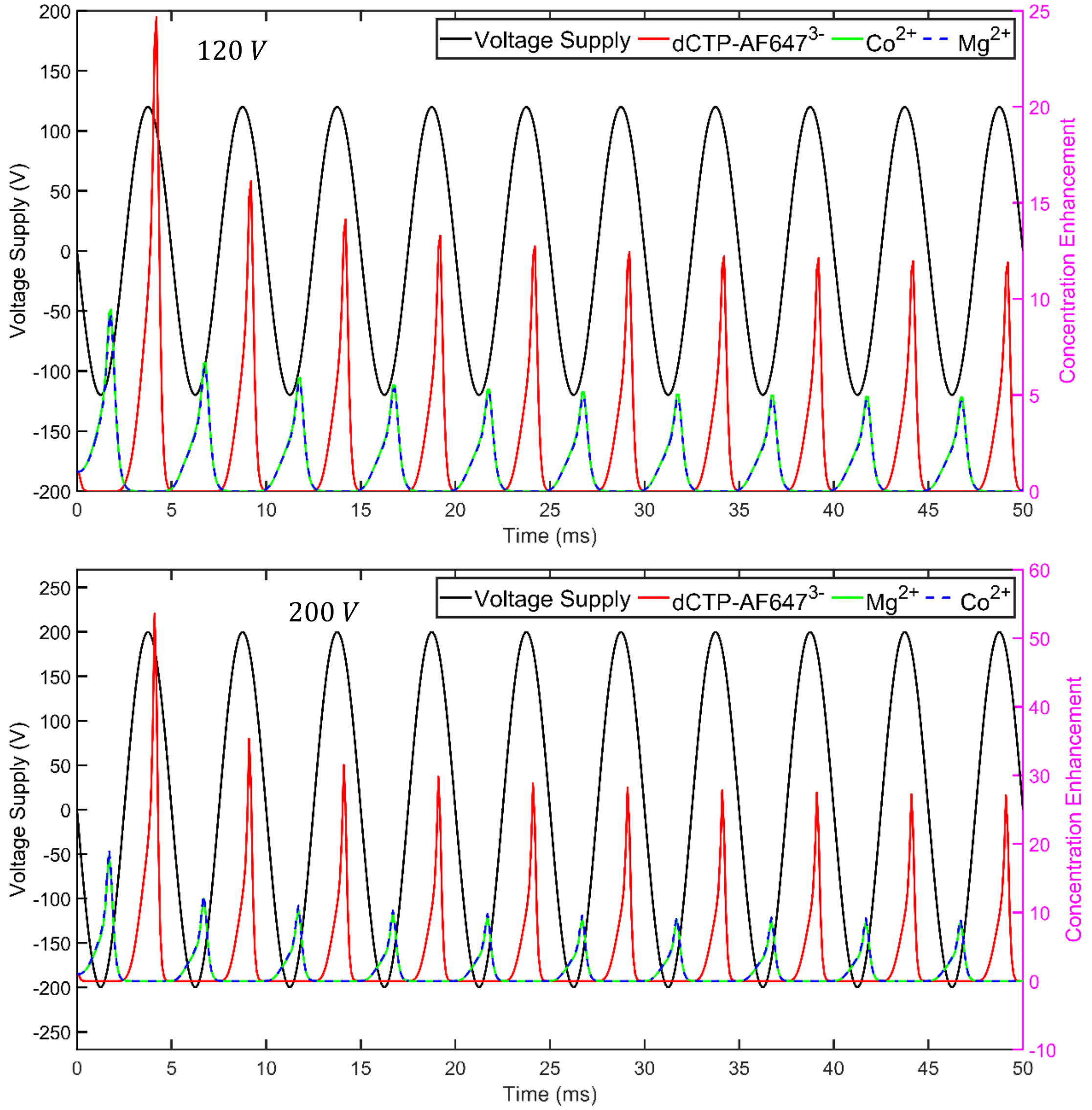

Supplementary Figure 17. Concentration enhancement of ionic reacting species during the A.C. supply cycle with varying supply voltage. (a) $120\,V$ (b) $200\,V$. Higher voltages can exert larger forces on the oppositely charged ionic species to induce higher average ionic concentration. At $120\,V$, $\langle ce_{dCTP-AF647}\rangle = 1.59$, $\langle ce_{Co}\rangle = 0.91$, $\langle ce_{Mg}\rangle = 0.89$. At $200\,V$, $\langle ce_{dCTP-AF647}\rangle = 2.78$, $\langle ce_{Co}\rangle = 1.23$, $\langle ce_{Mg}\rangle = 1.18$.

Supplementary References

S1. Bruus, H., Theoretical Microfluidics. Oxford University Press, Oxford (2008).

S2. Mugele, F., & Baret, J. C., Electrowetting: from basics to applications. *J. Phys. Condens. Matter*. **17**, R705-R774 (2005).

S3. Jackson, J. D., Classical Electrodynamics, 3rd edition. John Wiley & Sons, New York (1999).

S4. Griffiths, D. J., Introduction to Electrodynamics, 4th edition. Cambridge University Press, Cambridge (2017).

S5. Pethig, P., Dielectrophoresis: theory, methodology, and biological applications. John Wiley & Sons, Hoboken (2017).

S6. Padhy, P., Zaman, M. A., Jensen, M. A., & Hesselink, L., Dynamically controlled dielectrophoresis using resonant tuning. *Electrophoresis* **42**, 1079-1092 (2021).

S7. Jones, T. B., Electromechanics of particles. Cambridge University Press, Cambridge (2010).

S8. Jacqmin, D., Calculation of two-phase Navier-Stokes flows using phase-field modelling, *J. Comput. Phys.* **155**, 96-127 (1999).

S9. Berthier, J., Micro-drops and digital microfluidics. Elsevier, Oxford (2013).

S10. Clearco, PSF-1cSt pure silicone fluid octamethyltrisiloxane, **http://www.clearcoproducts.com/pdf/volatile/NP-PSF-1cSt.pdf** (2022).

S11. Ramos, A., Morgan, H., Green, N. G., Castellanos, A., A.C. Electrokinetics: A review of forces in microelectrode structures. *J. Phys. D: Appl. Phys* **31**, 2338-2353 (1998).

S12. Green, N. G., Ramos, A., Morgan, H., A.C. Electrokinetics: A survey of sub-micrometre particle dynamics. *J. Phys. D: Appl. Phys.* **33**, 632-641 (2000).

S13. Garde, R. J., Turbulent flow, New Age Science Limited, Kent (2010).

S14. Kirby, B. J., Micro and nanoscale fluid mechanics: transport in microfluidic devices. Cambridge University Press, Cambridge (2010).

S15. Michal, S., Hana, N., & Jan, K., The determination of viscosity at liquid mixtures-comparison of approaches, *AIP Conf. Proc.* **1889**, 020035 (2017).

S16. Cui, L., Holmes, D., & Morgan, H., The dielectrophoretic levitation and separation of latex beads in microchips. *Electrophoresis* **22**, 3893-3901 (2001).

S17. Torza, S., Cox, R. G., & Mason, S. G., Electrohydrodynamic deformation and bursts of liquid drops. *Philos. Trans. Royal Soc. A* **269**, 295-319 (1971).

S18. Mikkelsen, A., Kertmen, A., Khobaib, K., Rajnak, M., Kurimsky, J., & Rozynek, Z., Assembly of 1D granular structures from sulfonated polystyrene particles, *Materials* **10**, 1212 (2017).
