# Supplementary material for: Dielectrophoretic bead-droplet reactor for solid-phase synthesis
Source: Nat Commun. 2024 Jul 22;15:6159. doi: 10.1038/s41467-024-49284-z (PMC11263596; doi:10.1038/s41467-024-49284-z)
Supplement: Supplementary file 3 — Description of Additional Supplementary Files [file 41467_2024_49284_MOESM3_ESM.pdf]

Description of Additional Supplementary Files

Supplementary Movie 1: Encapsulation and Ejection of a Single Microbead into and out of a Single Microdroplet.

A single streptavidin coated polystyrene bead ($R_b = 3\ \mu m$) is encapsulated into the reagent microdroplet ($R_d = 25\ \mu m$) when the voltage supply (V_s) is ≈ 120 V and is ejected out of the microdroplet when the voltage supply (V_s) is ≈ 0.1 V.

Supplementary Movie 2: Microbead Encapsulation Slowed Down

Encapsulation of a streptavidin coated microbead (V_s) into a microdroplet ($R_d = 25\ \mu m$) at 1 frame per second to focus on the transition of the microbead into the microdroplet.

Supplementary Movie 3: Microbead Ejection Slowed Down

Ejection of a streptavidin coated microbead ($R_b = 3\ \mu m$) out of microdroplet ($R_d = 25\ \mu m$) at 1 frame per second to focus on the transition of the microbead out of the microdroplet.

Supplementary Movie 4: Shorter Encapsulation Time of Microbead in Microdroplet

Microbead remains encapsulated within the microdroplet for ≈ 4 s ($T_{ENC} = 4\ s$).

Supplementary Movie 5: Microfluidic Generation of Single Microdroplet

A single microdroplet is generated by a applying a pressure pulse of ≈ 10 mbar for a time period of ≈ 300 ms.

Supplementary Movie 6: Microfluidic Generation of Multiple Microdroplets

Two microdroplets are generated by a applying a pressure pulse of ≈ 20 mbar for a time period of ≈ 300 ms.

Supplementary Movie 7: Multiple Encapsulation and Ejection of Microbead from Microdroplet

A microbead can undergo multiple encapsulations and ejections necessary for multistep synthesis reactions.
