# Supplementary figures and images for: Dielectrophoretic bead-droplet reactor for solid-phase synthesis
Source: Nat Commun. 2024 Jul 22;15:6159. doi: 10.1038/s41467-024-49284-z (PMC11263596; doi:10.1038/s41467-024-49284-z)

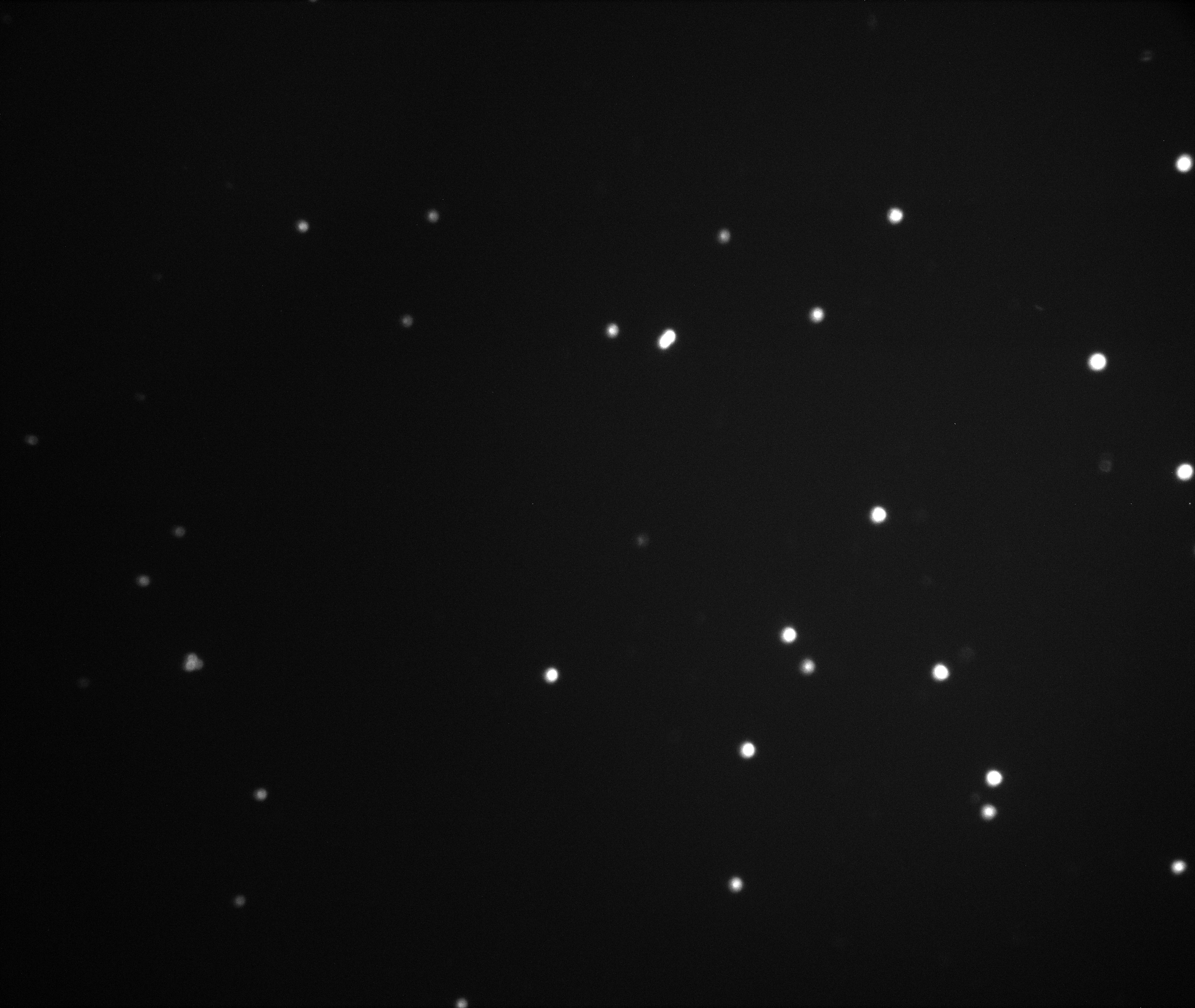

Supplement: Supplementary file 11 — Source Data [file 41467_2024_49284_MOESM11_ESM.zip › Codes_and_Data/Column_Fluorescence_Data_Extraction/im4_16.tif]
